# An integrated machine learning framework for TCM five-flavor classification based on E-tongue profiling and SHAP analysis

**DOI:** 10.1186/s13020-026-01399-9

**Published:** 2026-06-04

**Authors:** Ziang Li, Xianglong Meng, Lin Yang, Xiaoning Yan, Liangting Li, Yiwei Fu, Jingjing Tang, Shuosheng Zhang

**Affiliations:** 1 College of Chinese Materia Medica and Food Engineering, Shanxi University of Chinese Medicine, Jinzhong, 030619 China; 2 Key Laboratory of Traditional Chinese Medicine Processing in Shanxi Province, Jinzhong, 030619 China; 3 Traditional Chinese Medicine Processing and Inheritance Base, Shanxi University of Chinese Medicine, Jinzhong, 030619 China; 4 Key Research Laboratory of Processing and Innovation in Traditional Chinese Medicinal Materials, Shanxi University of Chinese Medicine, Jinzhong, 030619 China

**Keywords:** Electronic tongues, Five flavors, Machine learning, Heterogeneous stacking ensemble, Explainable artificial intelligence, SHAP analysis

## Abstract

**Background:**

The “five-flavor” (*wu wei*) classification is a core organizing principle in Traditional Chinese Medicine (TCM) pharmacology and quality control, yet its assessment still relies on subjective organoleptic evaluation, limiting standardization and reproducibility.

**Methods:**

Eighty-four processed herbal slices were analyzed using an electronic tongue to obtain multichannel sensor response profiles. Based on these data, we developed a dual-strategy ensemble learning framework in which a heterogeneous stacking model was trained for five-flavor identification while a voting ensemble was established in parallel for intensity grading. Model interpretability was evaluated using SHapley Additive exPlanations (SHAP) to quantify feature contributions and to relate prediction outcomes to specific electrochemical response patterns.

**Results:**

On the independent test set, the stacking ensemble showed moderate and flavor-dependent classification performance, with a macro-AUC of 0.876 and a balanced accuracy of 0.629. Discriminative performance was higher for Bitter and Sweet than for Sour, Salty, and Pungent. Full-stack SHAP analysis further identified P_13 and P_8 as the most influential features overall, with P_13 contributing mainly to the discrimination of bitter and pungent attributes, whereas P_8 was more strongly associated with sour, salty, and sweet characterization.

**Conclusions:**

This study demonstrates a data-driven workflow for digitizing TCM five-flavor assessment. Further validation with expanded sample sizes and chemical corroboration of sensor attributions is needed before operational deployment.

**Supplementary Information:**

The online version contains supplementary material available at 10.1186/s13020-026-01399-9.

## Introduction

Natural products derived from botanical and mineral sources serve as the historical foundation of pharmacotherapy [1]. Early medical systems ranging from Galenic medicine to Ayurveda relied on organoleptic evaluation to classify these complex materials [2–4]. In these traditions, taste functioned as a primary indicator of safety and therapeutic potential rather than a subjective descriptor [5]. Traditional Chinese Medicine (TCM) represents one of the longest continuously practiced examples of this empirical wisdom and serves an estimated 1.5 billion users globally [6]. The “five-flavor” (*wu wei*) theory acts as a core organizing principle within TCM pharmacology. This framework links the perceived taste of medicinal materials to characteristic physiological actions and therapeutic tendencies [7]. The five flavors consist of sweet (*gan*), bitter (*ku*), sour (*suan*), salty (*xian*), and pungent (*xin*). In clinical practice, these labels inform prescription design and processing decisions.

Modern receptor research provides a molecular foundation for this ancient taxonomy. Bitter perception involves the Taste 2 Receptors (TAS2Rs) family while sweet transduction and salty perception operate through specific heterodimers and ion channels [8–10]. These mechanistic insights confirm that flavor profiles reflect concrete chemical realities rather than purely subjective experiences. However, standard practice still relies on human organoleptic evaluation. This dependence introduces subjectivity and operator variability, creating a critical bottleneck for reproducible quality control.

Electronic tongue (E-tongue) systems offer an instrumental pathway to address this challenge. These devices employ arrays of cross-sensitive electrochemical sensors to generate multivariate response patterns [11]. Data-driven machine learning (ML) approaches can capture nonlinear patterns across complex natural product matrices without relying on single marker compounds [12]. When coupled with interpretability frameworks such as SHapley Additive exPlanations (SHAP), these models can move beyond mere prediction to provide feature-level attributions that are essential for regulatory acceptance [13]. E-tongue platforms have demonstrated utility for bitterness quantification in pharmaceutical formulations [14] and distinguished TCM materials with origins, processing methods, and storage conditions [15–17].

Despite the potential of E-tongue platforms, critical gaps impede their translation into a standardized characterization of the TCM five-flavor spectrum. First, current research predominantly targets bitterness quantification for taste masking [18, 19], leaving the holistic discrimination of the complete profile challenging. The “pungent” modality proves particularly difficult to characterize due to its chemical complexity [20, 21]. Moreover, conventional algorithms often struggle to capture the non-linear sensor responses induced by synergistic herbal matrices [22–24]. Second, a fundamental discrepancy remains unaddressed regarding the data structure. Most studies operationalize herbal analysis as supervised classification against predefined labels [25]. It remains unclear whether the intrinsic structure of these data naturally aligns with the empirical *wu wei*. Furthermore, the persistent opacity of models lacks feature-level interpretability required to link sensor signals with pharmacological plausibility.

To address these gaps, an integrated analytical framework coupling E-tongue profiling with heterogeneous stacking and voting ensembles, enhanced by multi-level SHAP interpretability, is presented in this study. A representative dataset was constructed based on the *Pharmacopoeia of the People's Republic of China* (CP, 2025 edition) to investigate whether instrumental signatures align with traditional flavor categories. Moving beyond single-model baselines, a two-tier stacking architecture was developed for five-flavor identification, alongside a soft-voting strategy for intensity discrimination. Crucially, SHAP analysis was employed to quantify feature-level contributions to model predictions, providing statistical attributions that generate testable hypotheses for subsequent chemical and electrochemical validation. This work aims to develop a reproducible, instrument-assisted proof-of-concept framework for TCM flavor assessment that produces chemically testable hypotheses compatible with traditional pharmacological reasoning.

## Materials and methods

**Fig. 1:**
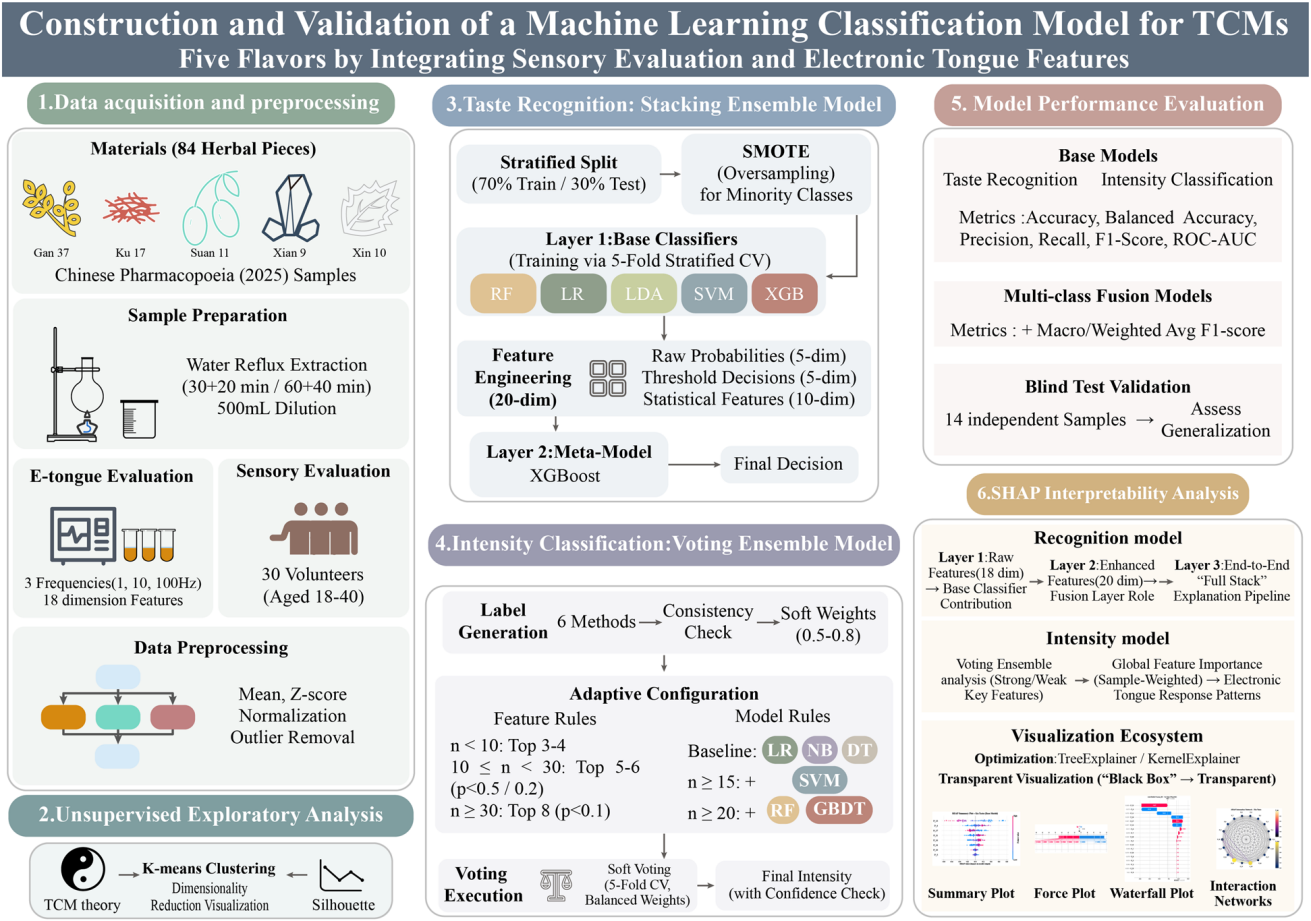
Overview of the experimental and analytical workflow. *Notes*: Processed herbal slices (n = 84) underwent sample preparation and parallel analysis via electronic tongue (18-dimensional features) and human sensory evaluation (30 trained panelists). ML models included a two-layer stacking ensemble for five-flavor recognition and an adaptive voting ensemble for intensity classification, with SHAP-based interpretability analysis

The overall experimental workflow is illustrated in Fig. 1. This pipeline encompasses the parallel acquisition of electronic tongue signals and human sensory data, followed by the development of hierarchical ensemble learning architectures—specifically stacking and voting strategies—to achieve interpretable flavor characterization.

### Materials

Prepared herbal slices were selected for this study based on the *Pharmacopoeia of the People's Republic of China* (CP, 2025 edition) and *Chinese Materia Medica*. Materials were excluded if they were classified as toxic or highly toxic, or if they exhibited strong intrinsic odors that could interfere with sensory assessment. A total of 84 processed herbal pieces were included, covering the five-flavor categories: sweet (*n* = 37), bitter (*n* = 17), sour (*n* = 11), salty (*n* = 9), and pungent (*n* = 10).

For algorithm development, samples were partitioned into a construction set of 71 samples and an external validation set of 14 samples. Glycyrrhizae Radix et Rhizoma, serving as the reference standard for sensory evaluation, was used exclusively for panelist training and calibration and its data were not used for model fitting or performance evaluation. Astragalus Radix and Scutellariae Radix were included in both the training and validation sets. These samples were drawn from the same batch but underwent independent extraction and measurement processes to ensure rigorous validation.

All samples were purchased from Beijing Tongrentang Shanxi Chain Pharmacy Co., Ltd. and were authenticated as qualified decoction pieces. Detailed information is provided in Supplementary Table S1.

### Sample preparation

Prepared herbal slices were soaked for 30 min and subjected to two-stage reflux extraction. General herbs were extracted for 30 and 20 min, while hard or tonic herbs underwent prolonged extraction (60 and 40 min) [26, 27]. Filtrates were combined and adjusted to 500 mL, corresponding to the mean standard dosages in the CP.

### Sensory evaluation and E-tongue measurement

Thirty trained volunteers (18–40 years) evaluated the samples, excluding individuals with oral/taste disorders or habits affecting taste (e.g., smoking) [28]. Panelists were trained on five basic tastes using reference standards: Glycyrrhizae Radix et Rhizoma (sweet) [29], Coptidis Rhizoma (bitter) [30], Mume Fructus (sour) [31], Zingiberis Rhizoma (pungent) [32], and NaCl (salty) [33]. Detailed information is provided in Supplementary Table S2. Samples were assessed randomly with a ≥ 3 min water-rinse washout to prevent carryover. Outliers were removed via Grubbs’ or Dixon’s tests, and missing values were median-imputed. After assessing normality (Shapiro–Wilk test) and variance homogeneity (Levene's test), Kruskal–Wallis H test followed by Dunn's post-hoc test with Bonferroni correction was used to compare taste categories due to unequal sample sizes and non-normal distribution (*p* < 0.05, SPSS 26.0).

Measurements were performed using an electronic tongue (Shanghai BosinTech, China) equipped with six metallic electrodes: gold (Au), silver (Ag), platinum (Pt), tungsten (W), titanium (Ti), and palladium (Pd). Measurements were conducted at 1, 10, and 100 Hz, generating 18-dimensional vectors (P_1 to P_18). A total of 436 profiles were obtained for 71 herbs (6–7 replicates). Data were averaged per herb, Z-score normalized, and integrated with sensory scores for analysis. The study was approved by the Shanxi University of Traditional Chinese Medicine Ethics Committee (No. 2025LL002), with written informed consent obtained from all participants.

### Unsupervised clustering and efficacy association analysis

To investigate the association between electronic tongue features and traditional efficacy, K-means clustering was applied to the standardized 18-dimensional features of the five medicinal flavor types. The optimal cluster number (*k*) was determined using a “data-driven and theory-guided” strategy: initial values were based on CP efficacy sub-categories (e.g., tonifying or diuretic for sweet flavor) and refined using Silhouette Coefficients and alignment with actual efficacies [34]. Finally, Principal Component Analysis (PCA), t-distributed Stochastic Neighbor Embedding (t-SNE), and Uniform Manifold Approximation and Projection (UMAP) were employed to reduce the 18-dimensional features to two dimensions for visualization (random state = 42).

### Taste recognition: stacking ensemble

71 samples were stratified and randomly partitioned into training (*n* = 49, 70%) and test sets (*n* = 22, 30%) at the material level to prevent data leakage from repeated measurements. To mitigate class imbalance (Sweet: 33, Bitter: 16, Sour: 7, Salty: 7, Pungent: 8), Synthetic Minority Over-sampling Technique (SMOTE) was applied to the training set with k_*neighbors* = min(5, *n*_*minority *_− 1) [35].

A two-layer stacking ensemble was constructed. Layer 1 comprised five heterogeneous binary classifiers optimized for specific taste profiles (Table 1). Layer 2 (the meta-model) utilized a 20-dimensional augmented feature vector derived from base learner outputs, consisting of: 5 class-specific posterior probabilities, 5 threshold-gated binary decisions, 5 basic statistics (global maximum probability, top-2 probability margin, probability standard deviation, dominant class index, and supra-threshold class count), and 5 advanced statistics (Shannon entropy, cumulative top-3 probability mass, maximum supra-threshold probability, peak-to-mean probability ratio, and interquartile range). Hyperparameters were optimized via fivefold stratified cross-validation targeting Receiver Operating Characteristic-Area Under the Curve (ROC-AUC), with classification thresholds tuned for F1 scores rather than the default 0.5. The meta-learner, trained on out-of-fold predictions to ensure validity, employed an eXtreme Gradient Boosting (XGBoost) multi-classifier (n_estimators = 120, max_depth = 4, learning_rate = 0.05) with automatic class weighting [36].

**Table 1:** Configuration of five base classifiers

Taste category	Algorithm	Feature selection	No. features
Sweet	Random forest	RFE	10
Bitter	Logistic regression	RFE	8
Sour	LDA	SelectKBest (F-test)	5
Salty	SVM	SelectKBest (MI)	5
Pungent	XGBoost	SelectKBest (MI)	8

### Intensity classification: voting ensemble

To convert continuous sensory intensities into binary labels (Weak/Strong), a multi-method consensus strategy was adopted [37]. The final threshold was defined as the median derived from six complementary methods: K-means (midpoint of centers), Gaussian Mixture Models (midpoint of component means), Kernel Density Estimation (local minimum), Decision Trees (information gain split) [38], Fisher LDA (variance ratio maximization) [39, 40], and Grid Search optimization (maximizing Silhouette scores). Consistency was evaluated using inter-method standard deviation. To mitigate subjective uncertainty, samples within a transition zone (± 0.2 from the threshold) were assigned dynamic weights (0.5–0.8) based on their distance to the boundary, further penalized by sensory evaluation variance and normalized (mean = 1.0).

The ensemble architecture followed a sample-size (*n*) adaptive principle to balance model complexity with data scarcity [41]. Feature selection criteria were dynamically adjusted: 8 features with significance *p* < 0.1 were selected for *n* ≥ 30; criteria were relaxed to 6 features with *p* < 0.2 for 20 ≤ *n* < 30, and 5 features with *p* < 0.5 for 10 ≤ *n* < 20. For extremely small samples (n < 10), the top 3–4 features ranked by F-scores were directly selected. All features were Z-score standardized.

The base model pool was similarly adaptive: Logistic Regression (LR), Naive Bayes (NB), and Decision Trees (DT) served as baselines, with Support Vector Machine (SVM) added for n ≥ 15, and Random Forest (RF) plus Gradient Boosting Decision Tree (GBDT) included for n ≥ 20 [42]. An equal-weight soft voting mechanism was implemented. All base models underwent hyperparameter optimization via fivefold stratified cross-validation, with macro-averaged F1-score used as the selection criterion to align with the class-imbalance-aware evaluation strategy, together with automatic class balancing (class_weight = 'balanced') [43, 44]. High-confidence predictions were flagged only when probabilities deviated by more than 0.2 from the decision boundary (0.5) [45].

### Model evaluation & SHAP interpretability

Model performance was evaluated using a multi-dimensional metric system. For binary tasks (the five base taste classifiers and intensity classification), accuracy, precision, recall, F1-score, and ROC-AUC were calculated [46, 47]. For the multi-class fusion layer, macro-averaged and weighted F1-scores were additionally computed. To account for class imbalance in both identification and intensity tasks, balanced accuracy (BA) was reported alongside standard accuracy as a primary performance metric. For binary classifiers, BA was defined as the arithmetic mean of per-class recall: BA = (Sensitivity + Specificity)/2, where Sensitivity (True Positive Rate) measures recall for the positive class and Specificity (True Negative Rate) measures recall for the negative class. For the multi-class stacking ensemble, BA was computed as the macro-average recall across all K classes: BA = (1/K) × Σ Recall_k. Unlike standard accuracy, BA is insensitive to class distribution and provides an unbiased performance estimate (random guessing yields BA ≈ 0.5), making it particularly informative for datasets with imbalanced class proportions. Generalization capability was assessed via blind testing on 14 independent samples excluded from training, comprising 6 single-label samples and 8 compound-flavor samples, organized as seven with six paired raw/processed herb specimens.

To elucidate decision mechanisms, SHAP was employed to quantify feature contributions [48]. For taste discrimination, a three-layer interpretability architecture was constructed: (1) analyzing the contribution of 18 raw features to the five base classifiers; (2) evaluating the 20-dimensional augmented features (probabilities and statistics) within the fusion layer; and (3) establishing an end-to-end pipeline to trace the decision chain from raw inputs to final outputs. For intensity classification, the voting ensemble was analyzed to identify key features distinguishing “Weak” from “Strong” grades, with global importance calculated via sample-size weighting to reveal universal response patterns. The accuracy of explanations was verified by a SHAP reconstruction error < 0.01 [49].

TreeExplainer or KernelExplainer were selected based on model type to optimize efficiency. Black-box transparency was achieved through Summary Plots (feature ranking/direction), Force Plots (individual contributions), Waterfall Plots (cumulative effects), and interaction networks. All analyses were implemented in Python 3.8 (scikit-learn 1.0.2, XGBoost 1.7.0, SHAP 0.41.0) with a fixed random seed (42) for reproducibility [50].

## Results

### Sensory dataset characteristics

**Fig. 2:**
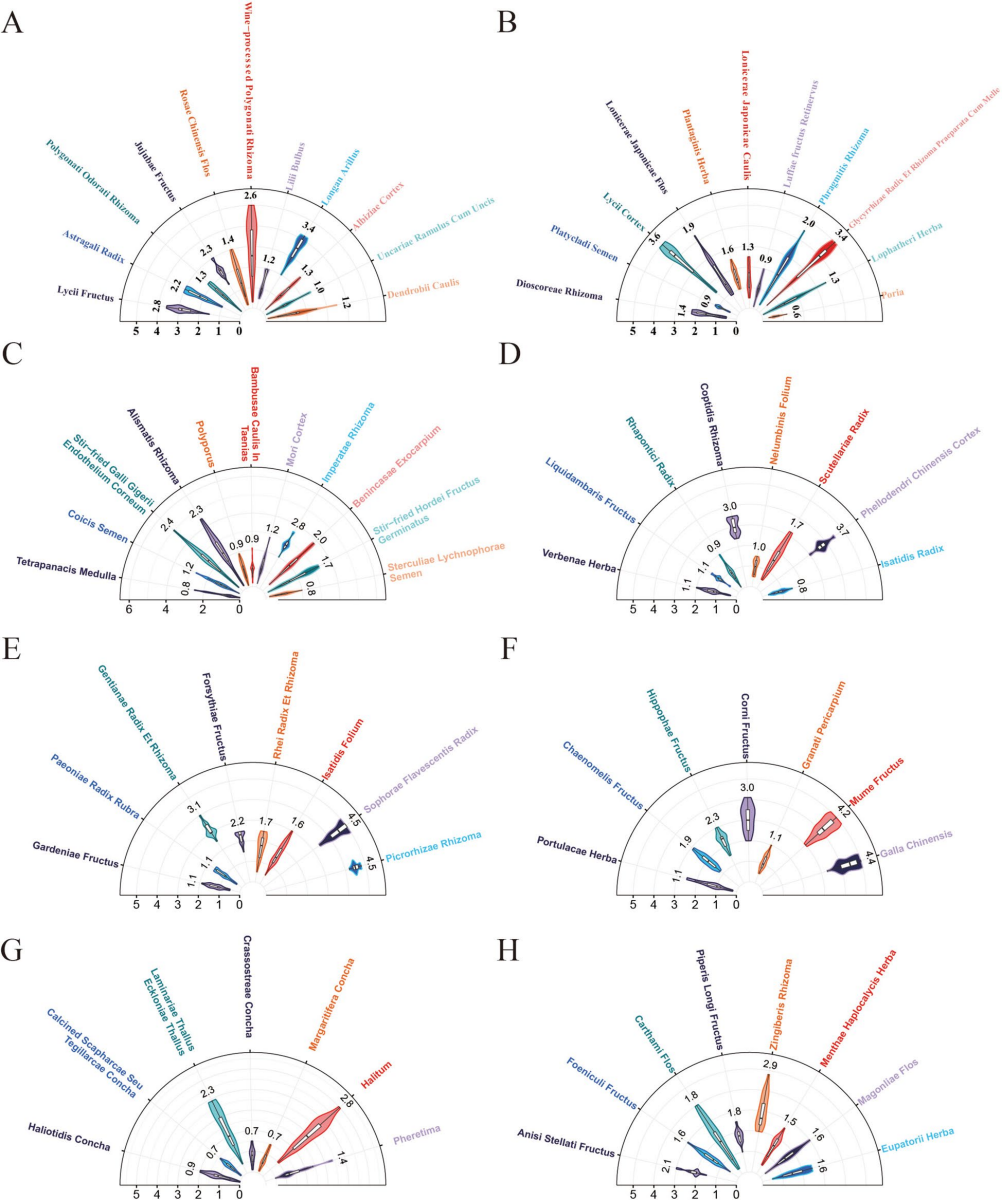
Sensory evaluation profiles of traditional Chinese medicines (TCMs) classified by the five-flavor theory. Semi-circular violin plots displaying the taste intensity scores of TCMs with **A**–**C** sweet (*gan*), **D**–**E** bitter (*ku*), **F** sour (*suan*), **G** salty (*xian*), and **H** pungent (*xin*) properties. The radial axis represents the sensory score, with larger values indicating stronger taste perception. Different colors denote individual TCM samples within each category

A total of 71 herbal slice samples were evaluated and categorized according to the CP. As detailed in Table 2, the dataset was predominantly composed of Sweet samples (n = 33, 46.5%), followed by Bitter (n = 16, 22.5%), Pungent (n = 8, 11.3%), Sour (n = 7, 9.9%), and Salty (n = 7, 9.9%). In terms of intensity, Sour samples exhibited the highest mean score (2.6 ± 1.4), whereas Salty samples were the weakest (1.4 ± 0.9). All categories displayed a right-skewed distribution (Mean > Median), indicating a prevalence of low-to-medium intensity samples (Fig. 2). The Pungent group demonstrated the highest assessor consistency (CV = 25.1%), while Salty and Bitter flavors showed substantial inter-individual variability (CV > 60%).

**Table 2:** Sensory evaluation dataset characteristics

Taste category	n	Mean ± SD	Median [IQR]	Range	CV (%)
Sweet	33	1.7 ± 0.8	1.4 [1.2–2.3]	0.6–3.6	47.9
Bitter	16	2.1 ± 1.3	1.7 [1.1–3.0]	0.8–4.5	62.1
Sour	7	2.6 ± 1.4	2.3 [1.5–3.6]	1.1–4.5	53.3
Salty	7	1.4 ± 0.9	0.9 [0.7–1.9]	0.7–2.8	64.1
Pungent	8	1.9 ± 0.5	1.7 [1.6–1.9]	1.5–2.9	25.1
*p*-value	–	0.223^a^	–	–	–

No significant differences were detected among taste categories at α = 0.05 level

*CV* coefficient of variation, *IQR* interquartile range

^a^Kruskal–Wallis H test (H = 5.70, df = 4, *p* = 0.223)

Despite the numerical trend where Sour intensity exceeded Salty by 89%, statistical analysis using the Kruskal–Wallis H test revealed no significant differences across the five categories (H = 5.70, df = 4, *p* = 0.223). This lack of statistical significance reflects the inherent heterogeneity of the dataset, characterized by unbalanced sample sizes (4.7:1 ratio) and high within-category variability, which obscured potential between-group distinctions.

### Unsupervised clustering and efficacy association

K-means clustering was applied to electronic tongue features, with cluster numbers (*k*) guided by TCM theoretical frameworks. The first two PCA components explained over 96% of the variance across all tastes (Table 3), confirming that the sensor array effectively captured the primary chemical variations.

**Table 3:** Summary of K-means clustering for five flavor categories

Taste category	k	Silhouette	PC1 + PC2 (%)	Cluster interpretation
Sweet	4	0.476	98.13	Tonifying, Harmonizing, Dampness-draining, Fluid-generating
Bitter	3	0.499	98.06	Heat-clearing/Damp-drying, Fire-purging, (Indigo-containing outlier)
Sour	3	0.484	98.41	Heat-clearing, Astringent, Strong-astringent
Salty	2	0.588	96.86	Liver-pacifying/Softening, Heat-clearing/Dissipating
Pungent	2	0.647	96.57	Dispersing/Blood-activating, Interior-warming

**Fig. 3:**
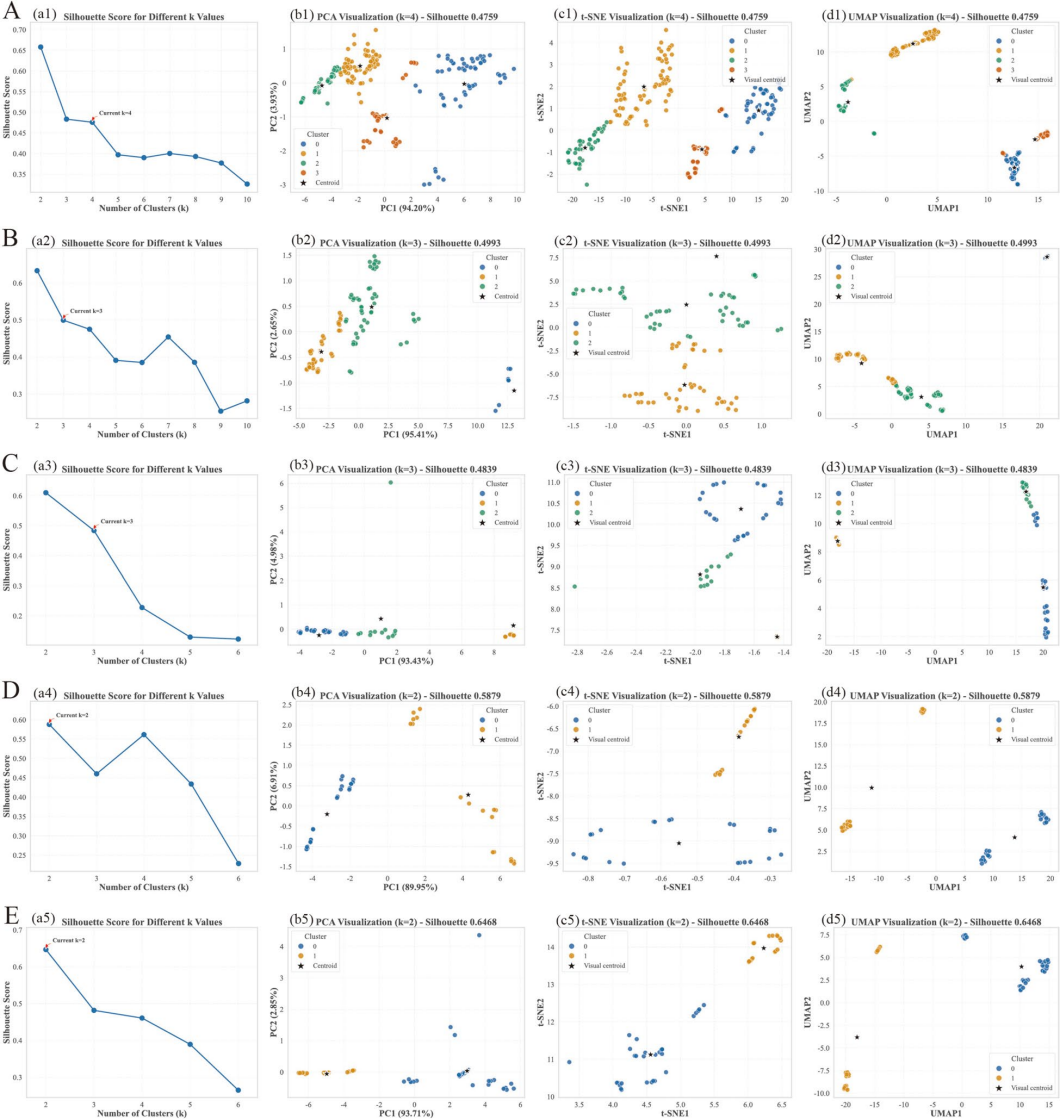
Clustering analysis of electronic tongue data from TCM decoction pieces grouped by five traditional tastes. **A** Sweet-taste group (k = 4), **B** Bitter-taste group (k = 3), **C** Sour-taste group (k = 3), **D** Salty-taste group (k = 2), and **E** Pungent-taste group (k = 2). For each taste group: (a1–a5) Silhouette score curves for different k values, with the optimal k marked by orange diamonds; (b1–b5) PCA visualization with cluster assignments and centroids; (c1–c5) t-SNE visualization; (d1–d5) UMAP visualization. Clustering was performed on 18 electronic tongue sensor features (P_1–P_18) using K-means algorithm. The silhouette scores for optimal clustering were 0.4759, 0.4993, 0.4839, 0.5879, and 0.6468 for sweet, bitter, sour, salty, and pungent groups, respectively

The clustering results aligned closely with traditional TCM functional classifications. Sweet samples (*k* = 4, Silhouette Coefficient [SC] = 0.476) formed four distinct groups corresponding to “Tonifying and Nourishing Yin” (Cluster 0: e.g., Astragali Radix), “Harmonizing and Moderating” (Cluster 1: e.g., Glycyrrhizae Radix et Rhizoma Praeparata cum Melle), “Draining Dampness” (Cluster 2: e.g., Poria), and “Promoting Fluid Production” (Cluster 3: e.g., Phragmitis Rhizoma), validating the theory that “Sweet can tonify, moderate, and harmonize” (Fig. 3A).

In the bitter group (*k* = 3, SC = 0.499), clustering revealed chemotaxonomic distinctions (Fig. 3B). Isatidis Folium formed an independent cluster, while Isatidis Radix appeared as an outlier. This separation is consistent with the organ-specific chemical signatures of *Isatis indigotica* Fort., Isatidis Folium is enriched in indole pigments (e.g., indigotin) [51], whereas the root is characterized by sulfur-containing glucosinolates (e.g., epigoitrin) [52, 53]. These distinct metabolite profiles are consistent with the divergent sensor responses, though the electrode-level basis for this association has not been characterized. The remaining samples clustered into “Clearing Heat and Drying Dampness” (Cluster 1, alkaloid-dominant, e.g., Coptidis Rhizoma) and “Clearing Heat and Purging Fire” (Cluster 2, e.g., Rhei Radix et Rhizoma), aligning with the efficacies of “drying” and “draining”, while the “Bitter can firm” function remained unverified due to the absence of representative samples.

Within the sour group (Fig. 3C), Portulacae Herba consistently formed an isolated cluster. Unlike typical sour herbs dominated by astringent tannins, Portulacae Herba is characterized by a high load of organic acids (citric, oxalic) and unique nitrogenous compounds such as oleraceins and betalains [54–56]. Overall, the enrichment of organic acids, oleraceins, and betalain pigments offers a chemically plausible explanation for the observed Portulacae Herba cluster separation, but targeted linking of specific metabolites to individual sensor channels will be needed to confirm mechanism. The remaining samples divided into a standard “Astringing and Binding” group (Cluster 1: e.g., Corni Fructus) and a “Potent Astringing” group (Cluster 2: e.g., Chaenomeles Fructus, Galla Chinensis), which exhibits stronger efficacy directionality.

Salty (Fig. 3D) and Pungent (Fig. 3E) categories achieved the highest silhouette coefficients (0.588 and 0.647, respectively), indicating uniform chemical profiles. Salty samples separated into “Calming Liver and Softening Hardness” (Cluster 0: minerals like Crassostreae Concha) and “Clearing Heat and Dissipating Nodules” (Cluster 1: e.g., Halitum). Pungent samples differentiated into “Dispersing and Invigorating Blood” (Cluster 0: e.g., Carthami Flos) and “Warming Interior and Dispelling Cold” (Cluster 1: e.g., Foeniculi Fructus).

### Five-flavor recognition via stacking ensemble

#### Base model performance

**Fig. 4:**
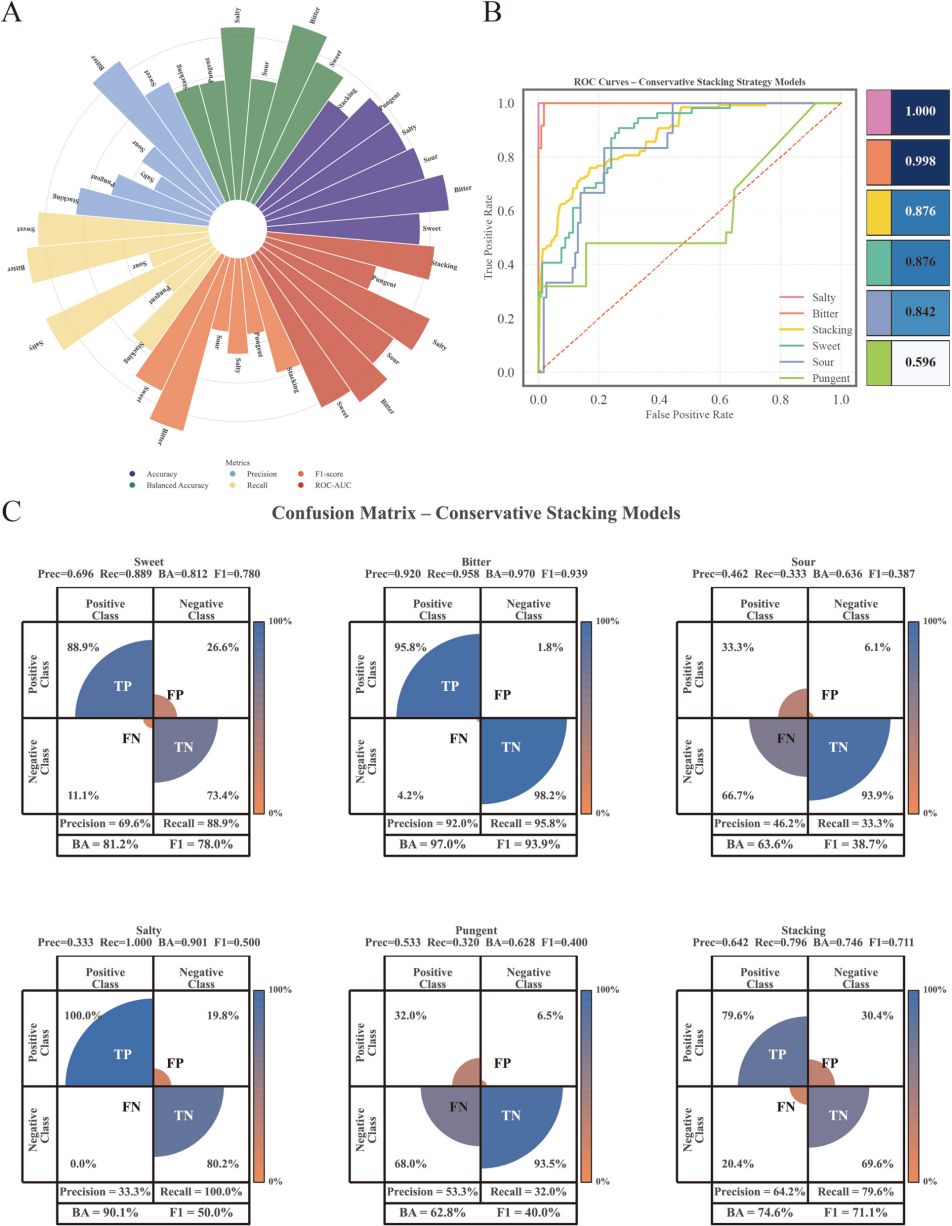
Comprehensive performance evaluation of the five taste-specific binary classifiers and the final Stacking ensemble. **A** Circular bar plots illustrating multi-dimensional evaluation metrics (Accuracy, Balanced Accuracy, Precision, Recall, F1-score, and AUC). Distinct colors within the bars correspond to different performance indicators as detailed in the legend. The Salty and Stacking models exhibit balanced metric profiles, whereas the Sour model shows suboptimal performance. **B** Receiver Operating Characteristic (ROC) curves comparing the diagnostic discriminative ability of the six models. Different colored curves represent distinct models. The Salty model achieved perfect discrimination (AUC = 1.000), while the Stacking ensemble demonstrated multi-class generalization compared to the single classifiers. **C** Confusion matrices visualizing the concordance between predicted labels and ground truth. The heatmap color gradient represents sample density, ranging from orange (low frequency) to blue (high frequency). High-intensity blue cells along the diagonal indicate accurate predictions, contrasting with the dispersed off-diagonal misclassifications observed in the Sour and Pungent modality. Panel 6 ("Stacking") displays the Stacking ensemble's performance on the Sweet class under a one-vs-rest formulation (BA = 0.746, F1 = 0.711), shown here for direct comparability with the Sweet base classifier in Panel 1; overall five-class performance of the ensemble (accuracy = 0.669, macro-F1 = 0.625, macro-BA = 0.629) is reported separately in Table 4.

To address the chemical diversity of TCM flavors, five heterogeneous binary classifiers were optimized using distinct algorithms and feature selection strategies (Fig. 4, Table 4). Performance varied significantly across categories, reflecting the inherent electrochemical heterogeneity of the taste modalities.

**Table 4:** Performance Metrics of Base Models and Stacking Model on Test Set

Model	Algorithm	Target flavor	Accuracy	Balanced accuracy	Precision	Recall	F1-score	AUC
Base-Sweet	Random Forest	Sweet (Gan)	0.797	0.812	0.696	0.889	0.780	0.8758
Base-Bitter	Logistic Regression	Bitter (Ku)	0.977	0.970	0.920	0.958	0.939	0.9977
Base-Sour	LDA	Sour (Suan)	0.857	0.636	0.462	0.333	0.387	0.8415
Base-Salty	SVM	Salty (Xian)	0.820	0.901	0.333	1.000	0.500	1.0000
Base-Pungent	XGBoost	Pungent (Xin)	0.820	0.628	0.533	0.320	0.400	0.5963
Stacking	XGBoost	Multi-class	0.669	0.629*	0.695*	0.629*	0.625*	0.8759*

*Macro-averaged metrics for multi-classification

The Bitter classifier (LR) achieved superior discrimination (ROC-AUC = 0.998), likely attributed to the strong, distinct electrochemical response of alkaloids (e.g., berberine) on specific sensor channels. Similarly, the Salty classifier (SVM) yielded a perfect AUC (1.000) due to the high ionic conductivity of salty compounds; however, its precision was lower (33.3%) as a conservative threshold was applied to prioritize sensitivity.

The Sweet classifier (Random Forest) maintained a balanced profile (F1 = 78.0%), consistent with the structural diversity of sweet constituents such as polysaccharides, glycosides, and amino acids. Conversely, the Sour classifier (LDA) exhibited a pronounced discrepancy between standard accuracy (0.857) and balanced accuracy (0.636, gap = 0.221), indicating that the high accuracy was substantially inflated by the majority class (non-Sour) and does not reflect genuine discriminative capacity for Sour samples (Sensitivity = 0.333). The signal overlap with other ionic modalities likely accounts for this low per-class recall. The Pungent classifier (XGBoost) faced the most significant challenges (AUC = 0.596). Critically, the standard accuracy of 0.820 is misleadingly inflated by class imbalance; the balanced accuracy of 0.628 (Sensitivity = 0.320, Specificity = 0.935) more faithfully represents the model’s true discriminative capacity, revealing that the classifier achieved its high overall accuracy primarily by correctly rejecting non-Pungent samples while failing to identify most actual Pungent instances. This limitation highlights a fundamental sensing principle: pungency is primarily a nociceptive sensation (pain) rather than a gustatory one, resulting in weaker electrochemical fingerprints for volatile oils or capsaicinoids on the electronic tongue.

#### Stacking ensemble performance

The stacking ensemble, utilizing an XGBoost meta-learner on 20 augmented features, achieved an overall accuracy of 66.9% (balanced accuracy = 0.629) and an ROC-AUC of 0.876 on the independent test set (n = 133) (Fig. 4, Table 4). The modest gap between accuracy and balanced accuracy (0.040) indicates that the multi-class ensemble is less susceptible to class-imbalance inflation than individual base models, though per-class recall varied substantially.

The ensemble approach yielded heterogeneous improvements. Bitter detection showed the strongest per-class performance (F1 = 0.86), while Sweet recognition was effectively preserved (F1 = 0.71). Notably, the Sour category saw a substantial leap in performance compared to its base model (F1 increased from 0.387 to 0.68; Recall from 33.3% to 78%). This suggests that the meta-learner successfully integrated orthogonal information from other classifiers to correct the conservative bias of the base LDA model.

However, challenges persisted for Salty and Pungent categories. The low precision in Salty predictions (40%) indicates that the meta-learner struggled to filter false positives generated by the high-recall base model. Similarly, the low recall for Pungent samples (28%) confirms that ensemble learning cannot fully compensate for the intrinsic lack of distinct sensor responsiveness to pungent compounds.

#### External validation

The external validation set (n = 14) was intentionally designed as a heterogeneous stress test, serving three purposes: (1) probing single-label generalization on architecturally compatible samples; (2) characterizing multi-label boundary conditions of the current single-label framework; and (3) tracking processing-induced flavor shifts across seven paired raw/processed herb specimens. Of the 14 samples, 8 (57.1%) carry compound-flavor profiles (multi-label, e.g., Crataegi Fructus: Sour/Sweet; Bupleuri Radix: Pungent/Bitter), while 6 (42.9%) are single-label samples architecturally compatible with the current framework. Because the current single-label architecture outputs exactly one predicted label per sample, any multi-label sample has a theoretical strict accuracy ceiling of 0% by construction. Results are accordingly presented below using a stratified evaluation framework.

For single-label compatible samples (n = 6), the strict accuracy was 33.3% (2/6). Astragali Radix and Astragali Radix Praeparata Cum Melle were correctly classified as Sweet. Two samples (Rehmanniae Radix and Rehmanniae Radix Praeparata) were predicted as Sour despite pharmacopeial Sweetlabels (Fig. S1). This discordance may reflect two non-exclusive factors: (i) direct organoleptic assessment by the authors confirmed sour-leaning sensory characteristics, suggesting that pharmacopoeial annotations conflate functional-efficacy attributes with sensory descriptors; and (ii) a systematic Sour over-prediction was independently observed in the multi-label sub-cohort for samples with high ionic conductivity, indicating limited sensor selectivity between acidic and ionic responses. Both mechanisms plausibly contribute, and these samples are flagged for targeted re-evaluation once sensor selectivity has been optimized.

Two samples (Scutellariae Radix and Wine-processed Scutellariae Radix) were genuinely misclassified as Pungent with low confidence scores (0.460 and 0.504, respectively), consistent with the known weakness of the Pungent base classifier (AUC = 0.596). Bupleuri Radix and Vinegar-processed Bupleuri Radix yielded a Label Recall of 0 (Fig. S2).

**Fig. 5:**
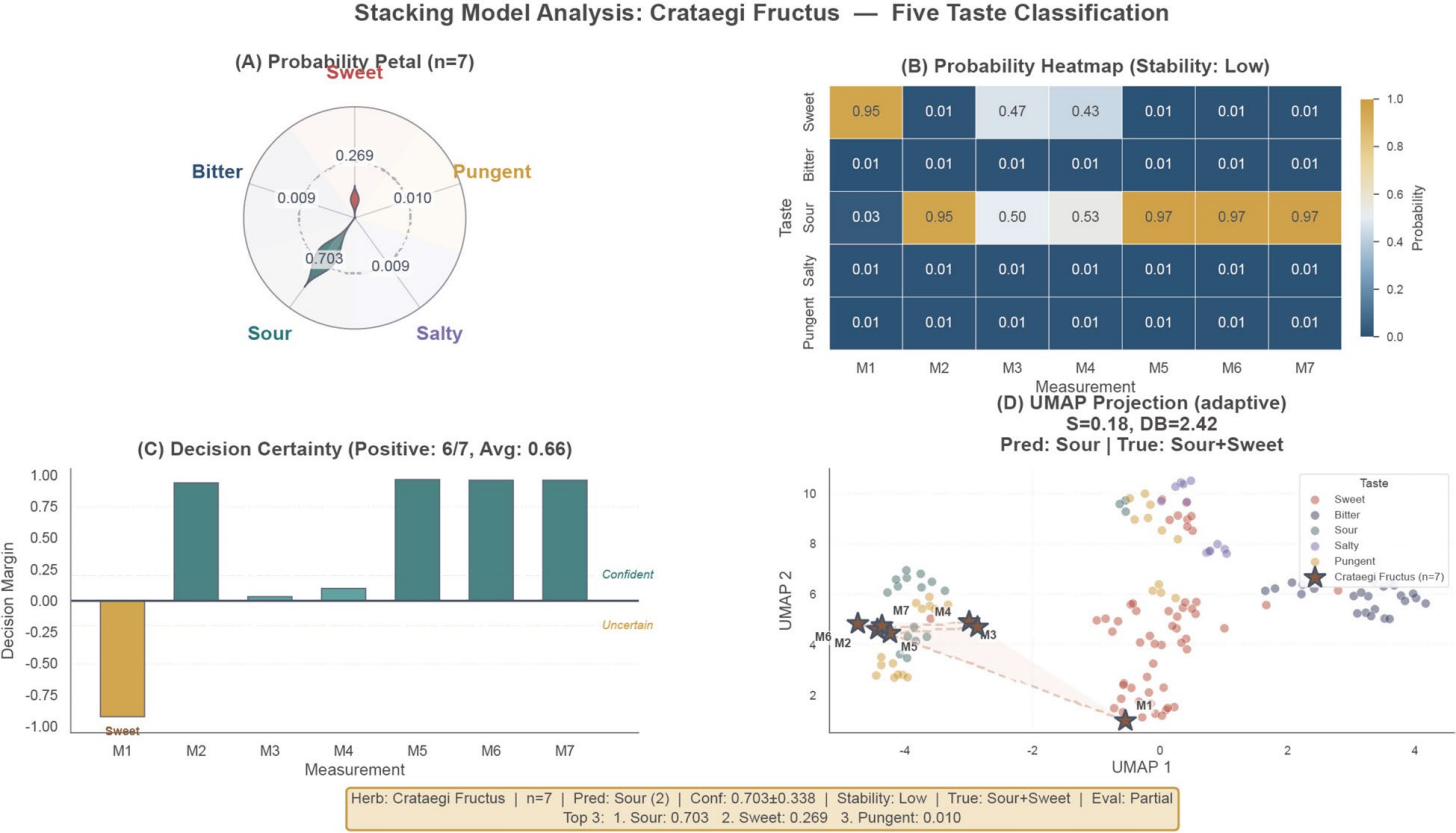
External validation of the stacking ensemble model using Crataegi Fructus as a representative case study. **A** Petal radar chart illustrating the predicted probability distribution across TCM flavor categories, identifying Sour as the dominant attribute. **B** Heatmap visualization of prediction scores for individual measurement replicates, demonstrating the consistency of the model across repeated samples. **C** Decision certainty analysis quantifying the confidence level of the predictions to assess classification reliability. **D** Scatter plot visualizing the distribution of Crataegi Fructus samples within the global classification landscape

The mean Label Recall across the 8 multi-label samples was 0.229. Using Crataegi Fructus as a representative case study (Fig. 5), the model assigned Sour as the dominant predicted attribute, and the processing series demonstrated a scientifically pattern: model confidence for Sour prediction decreased with increasing charring intensity (raw: 0.703 → lightly-fried: 0.881 → charred: 0.335), and the fully carbonized specimen (Crataegi Fructus Carbonisatus) was reclassified as Bitter with high confidence (0.941) (Fig. S3), consistent with the introduction of carbonization-derived bitter compounds. However, a systematic over-prediction of Sour flavor was identified, particularly for samples with high ionic conductivity (Natrii Sulfas series, confidence > 0.92)  (Fig. S4). This suggests insufficient sensor selectivity between acidic and strong ionic responses, representing a genuine limitation of the current sensor array configuration.

Overall, the external validation reveals that the current framework demonstrates primary-flavor identification within single-label compatible samples and provides interpretable tracking of processing-induced flavor transitions, while exposing two priority targets for future development: extension to a multi-label classification architecture and optimization of sensor selectivity for ionic versus acidic responses.

### Feature attribution via SHAP analysis

#### Base model feature attribution

**Fig. 6:**
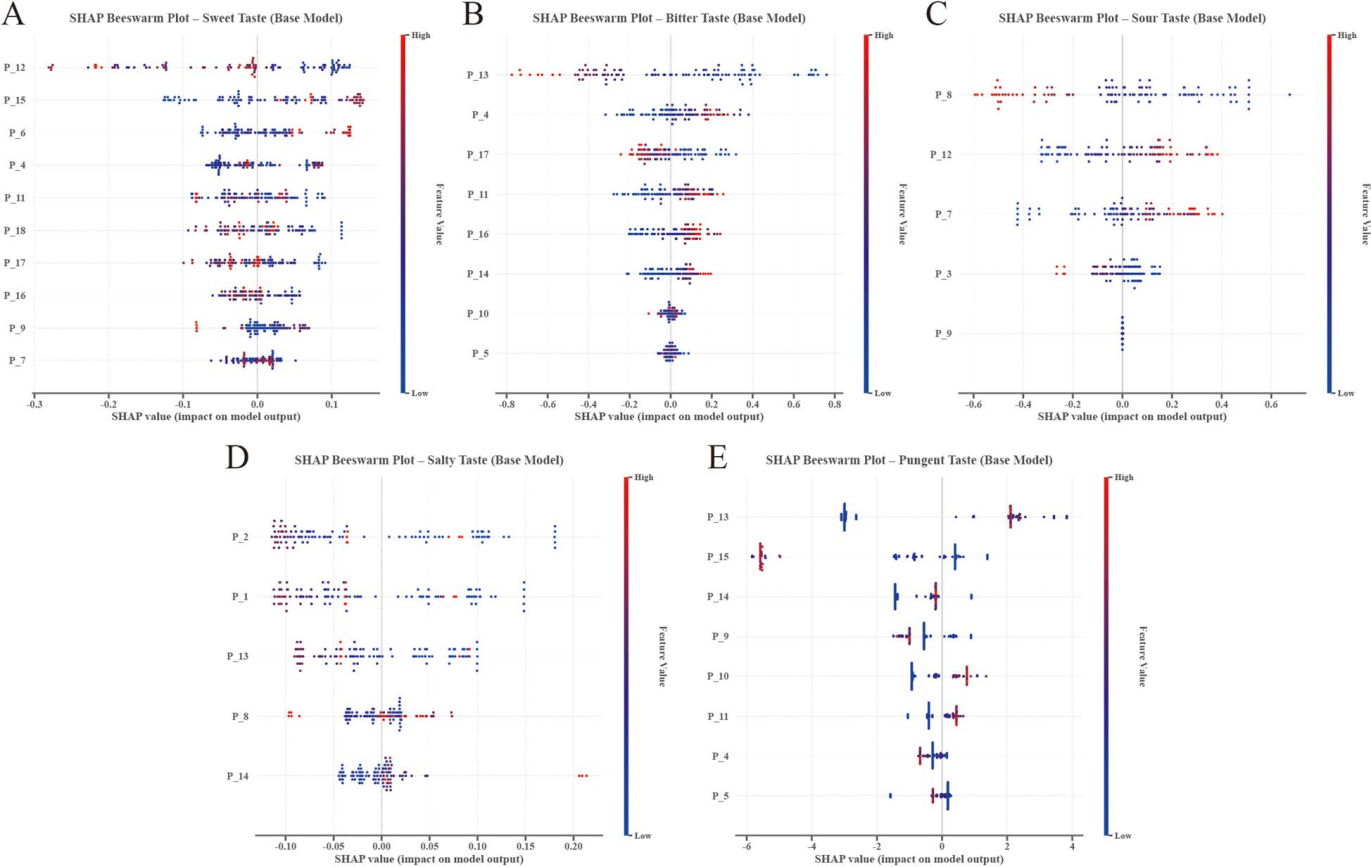
Base model feature attribution (**A**–**E**) visualized via beeswarm plots. The results reveal distinct sensor utilization patterns reflecting chemical heterogeneity; for instance, Bitter prediction is dominated by P_13, while Sweet recognition relies on a distributed profile (P_12, P_15, P_13), mirroring its complex chemical matrix

SHAP analysis was performed on the five binary base classifiers to quantify sensor contributions to taste prediction (Fig. 6). Each classifier exhibited a distinct feature utilization pattern, reflecting the chemical heterogeneity underlying different taste modalities.

In Bitter (*Ku*) recognition, sensor P_13 emerged as the dominant predictor (SHAP values: − 0.8 to + 0.8) with a strong positive correlation, indicating that high sensor responses drive bitter classification. P_4 and P_17 provided secondary contributions. This pattern is consistent with the statistical observation that bitter herbs are enriched in alkaloids and flavonoids. P_13 shows a strong statistical association with bitter classification in the model, a pattern that is congruent with the known alkaloid enrichment of bitter herbs, but the chemical basis of this sensor response requires independent validation. Conversely, Sweet (*Gan*) recognition displayed a distributed importance profile across P_12, P_15, P_13, P_4, and P_16, with no single feature dominating. This dispersion likely mirrors the complex chemical matrix of sweet herbs, which typically comprises diverse classes such as glycosides, polysaccharides, and amino acids.

Salty (*Xian*) recognition was characterized by P_2, which showed the highest importance but exhibited a complex non-linear relationship. The narrow SHAP range (− 0.10 to 0.20) suggests the SVM model relies on subtle variations in sensor response, consistent with the specific ionic conductivity of salty compounds. Pungent (*Xin*) prediction was driven by P_13 and P_15, which showed the largest SHAP magnitude (− 6 to + 4) among all base models. This indicates that pungent compounds generate distinct electrochemical signals strongly captured by these specific channels.

Cross-modality comparison identified P_13 as a universal discriminant, consistently ranking highest for Bitter (1st), Salty (3rd), and Pungent (1st) prediction. In contrast, P_8 displayed specificity for both Sour and Salty tastes, whereas P_2 was exclusively critical for Salty recognition. This differential feature utilization validates the rationale behind the heterogeneous ensemble approach (Table 5).

**Table 5:** Base model feature importance summary

Taste	Model	Top 3 features	SHAP range
Sweet (*Gan*)	Random forest	P_12, P_15, P_6	− 0.3 to + 0.1
Bitter (*Ku*)	Logistic regression	P_13, P_4, P_17	− 0.8 to + 0.8
Sour (*Suan*)	LDA	P_8, P_12, P_7	− 0.6 to + 0.7
Salty (*Xian*)	SVM	P_2, P_1, P_13	− 0.10 to + 0.20
Pungent (*Xin*)	XGBoost	P_13, P_15, P_14	− 6 to + 4

#### Meta-model and full-stack analysis

**Fig. 7:**
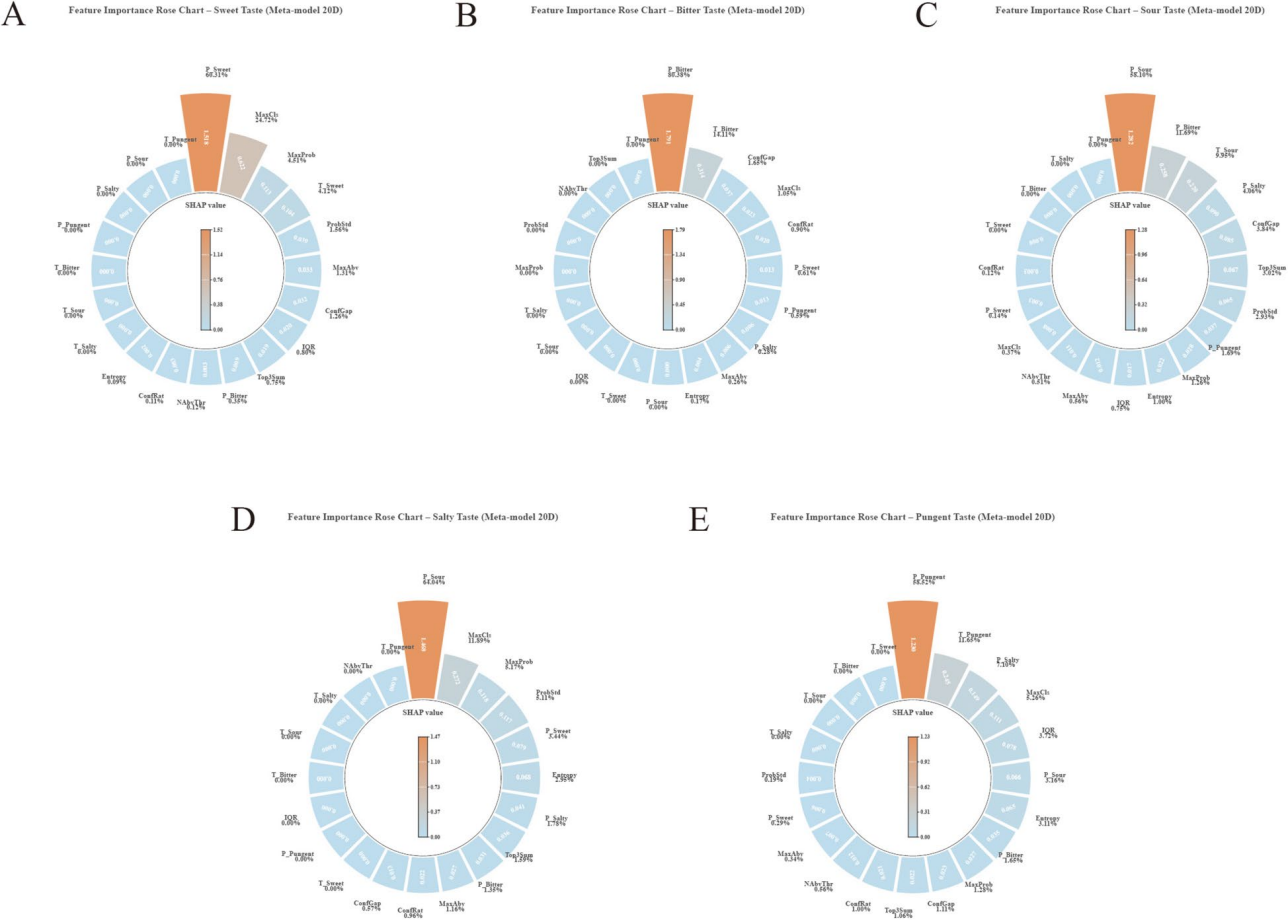
Polar bar charts (**A**–**E**) illustrating the feature importance of the 20-dimensional meta-layer. Probability-based features generally dominate the meta-learner’s decision; notably, the Salty model (b4) exhibits a unique inverse dependence on Sour probability (*P*_*Suan*_), indicating the model learns cross-taste exclusion logic

The meta-model, constructed upon 20-dimensional engineered features (comprising 5 probabilities, 5 threshold decisions, and 10 statistical derivatives), elucidated a hierarchical pattern of feature utilization (Fig. 7). Probability features dominate in all taste types (mean SHAP range: 0.5–2.5), followed by threshold decisions (0.1–0.5), while statistical derivatives contribute very little (< 0.1).

Notably, the prediction of Salty taste exhibited a distinct pattern wherein P_Sour (Sour probability), rather than P_Salty (Salty probability), emerged as the pivotal feature, displaying an inverse correlation where low sour probability strongly facilitated Salty classification. This suggests that the meta-model has learned to leverage ensemble-level interactions transcending single-taste probabilities, a phenomenon likely attributable to the diminished probability variability resulting from the near-perfect performance of the base models (AUC = 1.0).

**Fig. 8:**
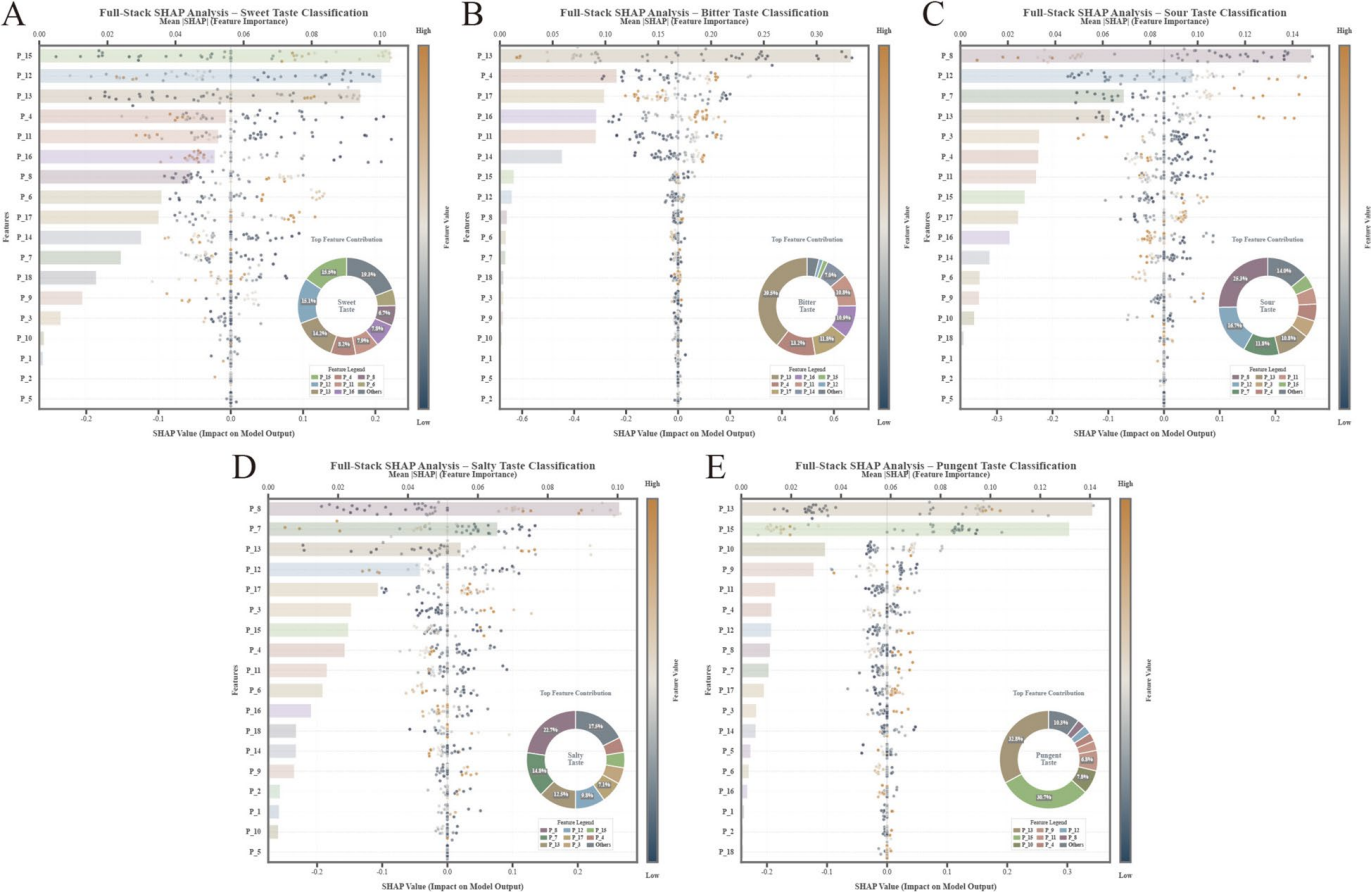
Full-stack global feature importance analysis (**A**–**E**). A composite visualization (integrating beeswarm plots, absolute importance bar charts, and relative contribution donut charts) is employed to trace the end-to-end influence of the original 18 sensors on the final classification. The analysis identifies P_13 as the globally most influential feature (weighted mean SHAP = 0.14), followed by P_8 and P_15. The global ranking designates P_13 and P_8 as universally significant sensors across multiple tastes

A full-stack end-to-end SHAP analysis directly traced the influence of 18 sensor features to the ultimate classification (Fig. 8, Table 6). P_13 was identified as the globally most influential feature (weighted mean SHAP = 0.14), followed by P_8 (0.065), P_15 (0.060), and P_12 (0.055). The global ranking designated P_13 and P_8 as universally significant sensors.

**Table 6:** Full-stack global feature importance (Top 5)

Rank	Feature	Weighted mean |SHAP|	Primary contribution
1	P_13	0.140	Bitter (1st), Pungent (1st), Salty (3rd)
2	P_8	0.065	Sour (1st), Salty (1st), Sweet (1st)
3	P_15	0.060	Sweet (2nd), Pungent (2nd)
4	P_12	0.055	Sweet (1st), Sour (2nd)
5	P_4	0.045	Bitter (2nd), Sweet (4th)

**Fig. 9:**
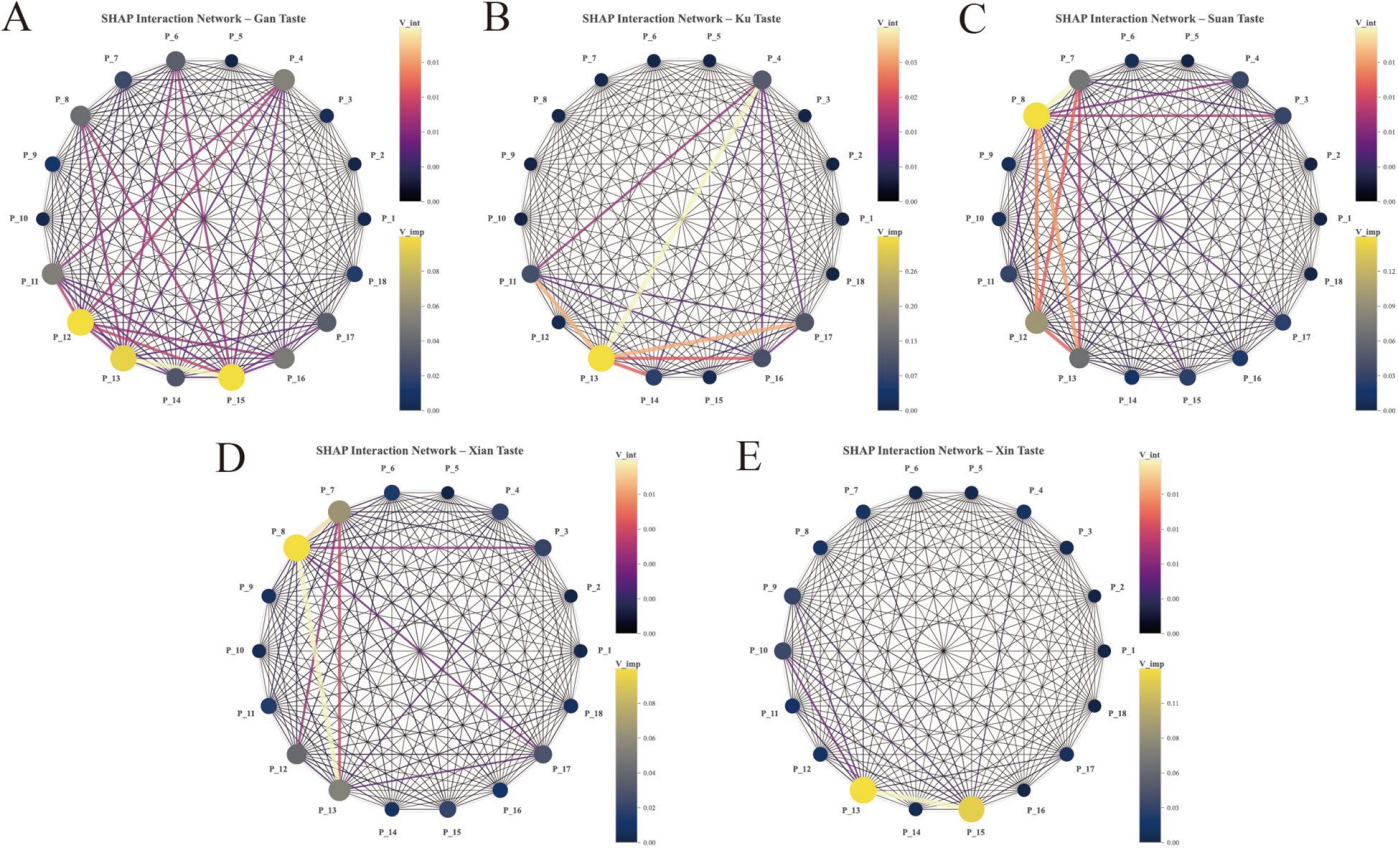
Visualization of taste-specific feature interaction networks and topological architectures. The network graphs illustrate the dependency structures between sensors derived from SHAP interaction values (*V*_*int*_), where nodes represent features and edges denote the strength of synergism. **A** Sweet: Displays a polycentric architecture with feature importance distributed among P_12, P_13, and P_15, reflecting the complex chemical matrix of sweet herbs. **B** Bitter: Exhibits a single-hub hierarchical structure anchored by P_13, with the P_13-P_4 link interaction (*V*_*int*_ = 0.03). **C**, **D** Salty and Sour: Manifest a distinct hub-and-spoke pattern centered on the pivotal P_8 **E** Pungent: Presents a dual-hub sparse structure, indicating that classification relies predominantly on independent feature contributions rather than complex inter-sensor synergism

Feature interaction network analysis unveiled taste-specific topological architectures (Fig. 9). Bitter taste exhibited a single-hub hierarchical structure anchored by P_13, with the P_4 – P_13 interaction proving the most stable (*V*_*int*_ = 0.03). Sweet taste displayed a polycentric architecture, with feature importance distributed among P_12, P_13, and P_15. Salty and Sour tastes manifested a hub-and-spoke pattern centered on P_8. Pungent taste presented a dual-hub sparse structure, indicating that classification relies predominantly on independent feature contributions rather than complex synergism.

#### Single-sample interpretation

**Fig. 10:**
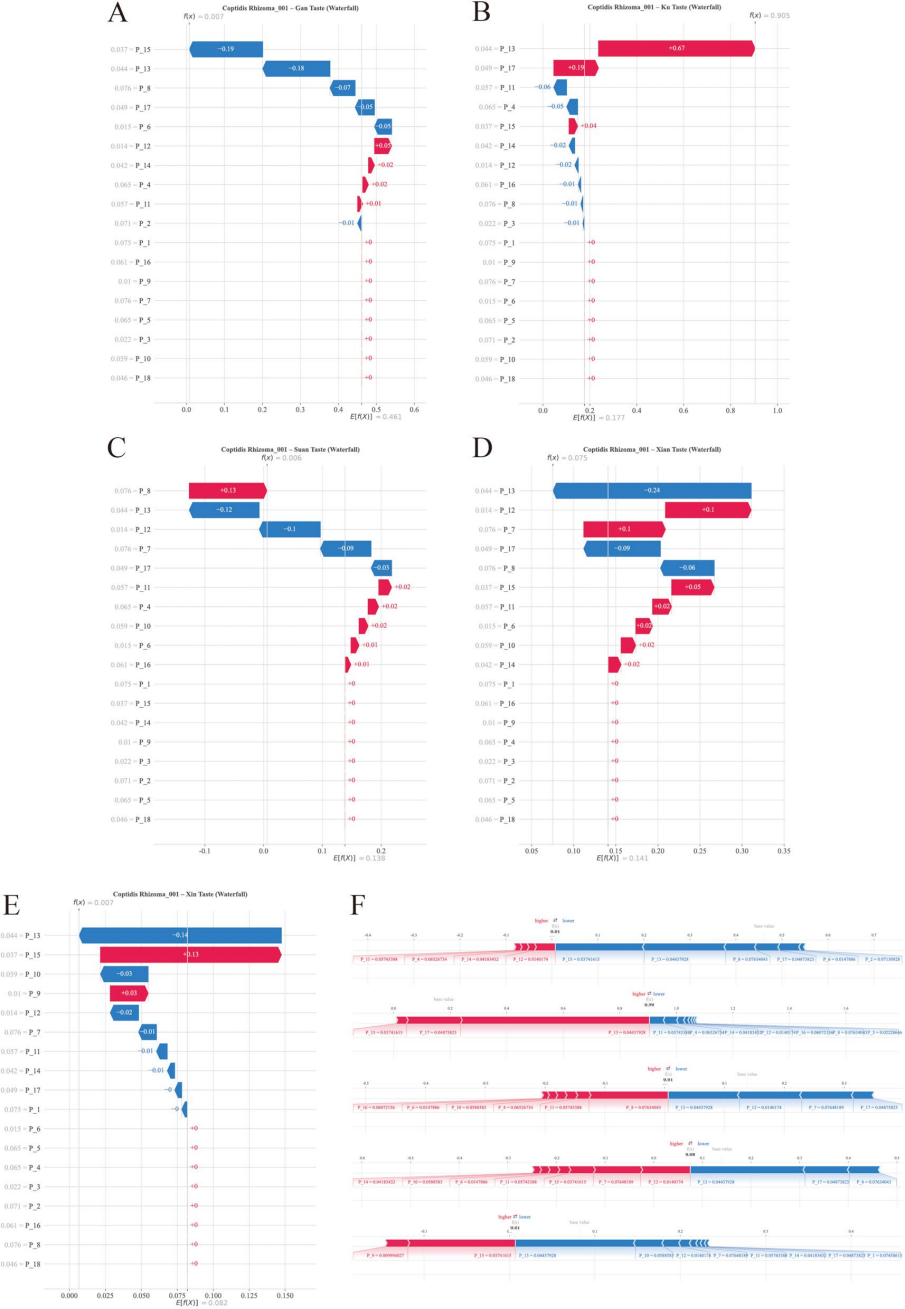
Individual-level feature attribution patterns revealed by SHAP waterfall plots using Coptidis Rhizoma and Phellodendri Chinensis Cortex as representative Bitter herbs. **A**, **C**–**E** Plots for non-target tastes (Sweet, Sour, Salty, Pungent) demonstrate the model’s suppression pattern in feature attribution. The probabilities are effectively attenuated to near-zero levels. **B** The waterfall plot for Bitter classification illustrates a decisive probability shift from the baseline expectation (*E[f(X)]* = 0.177) to the final prediction (*f(X)* = 0.905). **F** SHAP force plot visualizing Phellodendri Chinensis Cortex  prediction dynamics. The plot illustrates the “tug-of-war” between features, tracking the shift from the baseline expectation to the final model output

To examine the feature attribution pattern at the individual level, SHAP waterfall plots were generated utilizing Coptidis Rhizoma as a representative Bitter herb known for its high berberine content (Fig. 10). Regarding Bitter taste classification, the model elevated the predicted probability to 0.905 from a baseline expectation of *E*[*f*(*X*)] = 0.177, yielding a net positive shift of + 0.728. P_13 served as the primary driver, contributing + 0.67 (accounting for 92% of the total positive contribution), with ancillary support provided by P_17 and P_15 (+ 0.04).

Concurrently, the model effectively attenuated non-Bitter predictions: Sweet probability plummeted from 0.461 to 0.007, driven by P_15 (− 0.19) and P_13 (− 0.18); Sour probability declined to 0.006, suppressed by P_13 (− 0.12) and P_12 (− 0.10); and Pungent probability reached a nadir of 0.007, with P_13 (− 0.14) acting as the dominant inhibitory factor.

This dualistic contribution profile—wherein P_13 simultaneously bolstered Bitter classification (+ 0.67) while suppressing Sweet (− 0.18), Sour (− 0.12), Salty (− 0.24), and Pungent (− 0.14) probabilities—suggests that this feature captures an electrochemical response pattern that is statistically associated with the alkaloid-enriched chemical profiles characteristic of bitter herbs.

The consistency of SHAP patterns observed between Coptidis Rhizoma and Phellodendri Chinensis Cortex (another berberine-rich herb) further suggests that the model captures reproducible electrochemical response patterns shared by chemically related herbs, rather than sample-specific artifacts, although the underlying chemical basis awaits targeted analytical confirmation.

### Intensity classification via voting ensemble

#### Threshold optimization and boundary definition

**Fig. 11:**
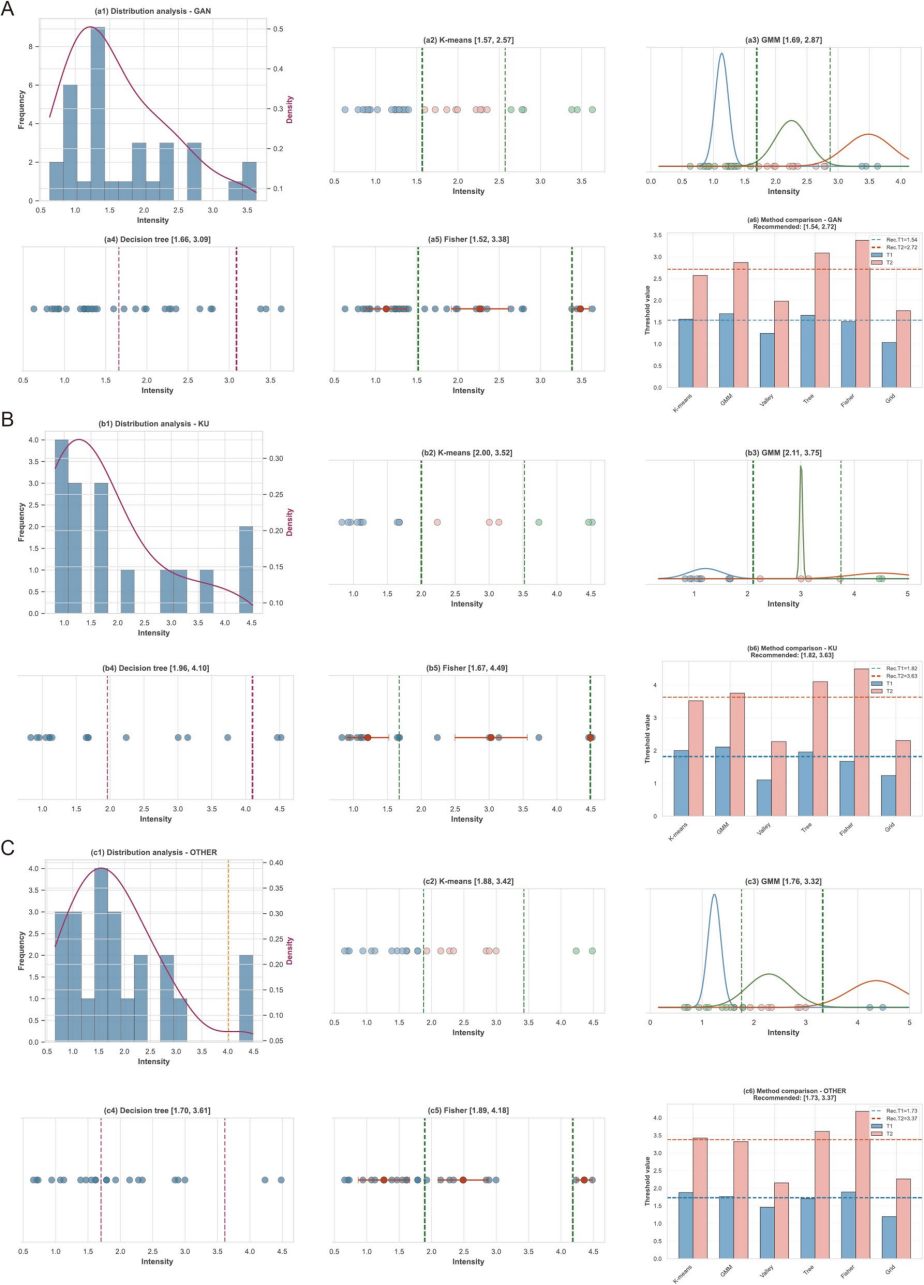
Multi-method threshold optimization analysis for binary sensory classification. Notes: The analysis is stratified by sensory attribute categories: **A** Gan (Sweet) data (panels a1–a6); **B** Ku (Bitter) data (panels b1–b6); and **C** Other attributes including Suan (Sour), Xin (Pungent), and Xian (Salty) (panels c1–c6). Within each category, the sub-panels illustrate the consensus decision process: (1) Probability density distribution (KDE) showing peak-valley structures. (2–5) Visualization of decision boundaries derived from K-means, GMM, Decision Trees, and Fisher Discriminant Analysis. (6) Summary of thresholds across all methods. The high variability in the upper threshold (*T*_*2*_*,* red bars) contrasts with the stability of the lower threshold (*T*_*1*_*,* blue bars), supporting the adoption of a robust binary classification scheme. The final consensus boundary (dashed line) is defined as the median derived from the multi-method strategy. Panel C represents the pooled exploratory analysis for the small-sample categories only; the final independent voting models used taste-specific thresholds, as reported in Table 7.

Objective intensity classification boundaries were determined through a multi-method quantitative analysis (Fig. 11). The initial exploration for a ternary classification scheme (Weak/Medium/Strong) revealed significant methodological divergence at the upper decision boundaries (*T*_*2*_). The inter-method standard deviation (SD) for *T*_*2*_ ranged from 0.58–0.84 across flavor categories (e.g., Bitter *T*_*2*_ SD = 0.84), indicating a lack of objective consensus for defining a distinct “Medium” intensity class.

In contrast, the lower binary thresholds (*T*_*1*_) distinguishing low-intensity signals from significant sensory responses demonstrated superior consistency, with SD values ranging from 0.24 to 0.38. The binary partition was further validated by Fisher Discriminant Analysis, which yielded high variance ratios (14.16–15.04), confirming that the binary scheme captures the primary discriminative structure of the data.

Consequently, a binary classification scheme (Weak vs. Strong) was adopted utilizing *T*_*1*_ as the cutoff. The final adaptive thresholds were established based on the median values of the six-method consensus: 1.54 for Sweet (*Gan*) and 1.82 for Bitter (*Ku*). Due to limited sample sizes (n < 10) for Sour, Salty, and Pungent samples, these categories were aggregated into a unified class with a consensus threshold of 1.73. To address the classification uncertainty observed in the Kernel Density Estimation (KDE) transition zones, a soft boundary margin of ± 0.2 intensity units was implemented. Samples falling within these regions (e.g., 1.34–1.74 for Sweet) were assigned dynamic weights (0.5–0.8) during the ensemble training to mitigate overfitting to ambiguous labels.

#### Classification performance

**Fig. 12:**
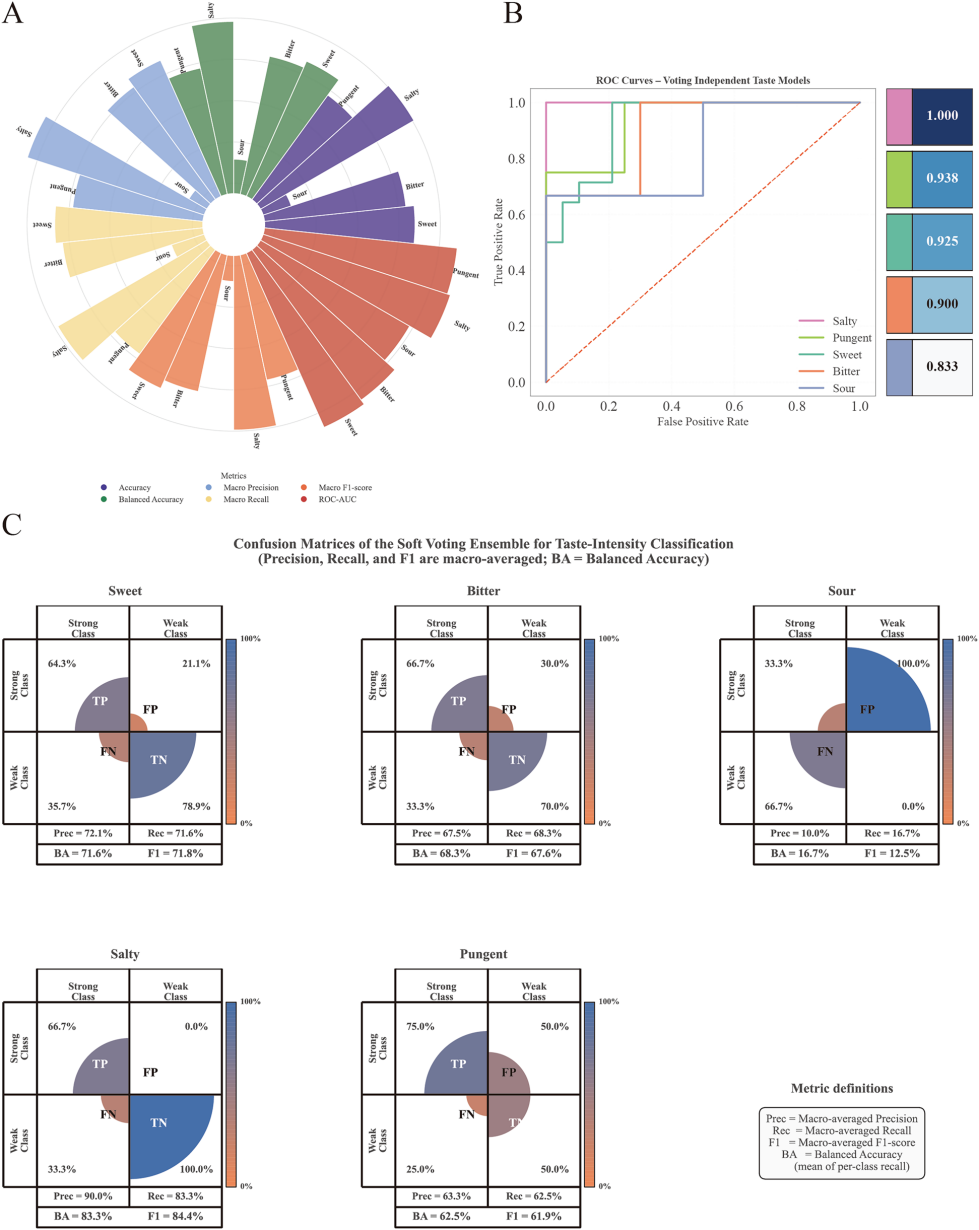
Comprehensive performance evaluation of the Voting Ensemble classification strategy independently constructed for five TCM taste modalities. *Notes*: All performance metrics were derived using Stratified 5-Fold Cross-Validation to ensure statistical robustness against sample size variations. **A** Circular bar plots illustrating multi-dimensional evaluation metrics (Accuracy, Balanced accuracy, Macro Precision, Macro Recall, Macro F1-score, and AUC). The visualization highlights the performance consistency of the Voting Ensemble strategy across the five specific taste categories (Sweet, Bitter, Sour, Salty, and Pungent). **B** ROC curves comparing the diagnostic discriminative ability of the five taste-specific models. The curves demonstrate the models' sensitivity and specificity in distinguishing Weak vs. Strong intensity signals, reflecting the varying classification difficulty across different flavor profiles. **C** Confusion matrices for each taste category visualizing the concordance between predicted labels and ground truth. The heatmap color gradient represents sample density, transitioning from orange (misclassification/low frequency) to blue (correct prediction/high frequency). The high concentration of samples along the diagonal axis confirms the effectiveness of the soft-voting ensemble strategy and adaptive thresholding mechanism

The soft voting ensemble exhibited distinct performance variations across different taste modalities (Table 7, Fig. 12). Salty taste achieved superior results (Accuracy = 85.7%, Macro F1 = 0.844, AUC = 1.000) despite the limited sample size (*n* = 7). This success was attributed to highly discriminative features (P_7, P_8, P_9; all *p* < 0.01) that effectively captured strong ionic sensor responses. Sweet taste demonstrated adequate performance (Accuracy = 72.7%, Macro F1 = 0.718, AUC = 0.925) with a balanced precision-recall trade-off, validating the efficacy of the ensemble approach given sufficient training data (*n* = 33). Bitter and Pungent tastes yielded acceptable performance (Accuracy: 62.5–68.8%, Macro F1: 0.619–0.676), where single decision trees occasionally matched or outperformed the ensemble under moderate sample size conditions. Conversely, Sour taste classification was essentially non-functional (Accuracy = 14.3%, Balanced Accuracy = 0.167, Macro F1 = 0.125, Generalization Gap = 80%). The balanced accuracy of 0.167 falls well below the random-chance baseline of 0.5, confirming that the model possesses no meaningful discriminative ability for Sour intensity grading. This failure was ascribed to a convergence of three factors: the minimal sample size (*n* = 7, with only 4 Weak and 3 Strong instances, far below the minimum required for reliable cross-validated evaluation); a lack of statistically significant discriminative features (all *p* > 0.69); and high intra-class variance (CV = 53.3%), which collapses the separation between Weak and Strong distributions. Given these constraints, the Sour intensity model should be regarded as a null result rather than a viable classifier.

**Table 7:** Performance of soft voting ensemble for intensity classification

Taste	n	Threshold	Features	Accuracy	Balanced Accuracy	Macro Precision	Macro Recall	Macro F1	AUC
Sweet	33	1.62	8	0.727	0.716	0.721	0.716	0.718	0.925
Bitter	16	1.87	5	0.688	0.683	0.675	0.683	0.676	0.900
Sour	7	2.49	3	0.143	0.167	0.100	0.167	0.125	0.833
Salty	7	1.19	3	0.857	0.833	0.900	0.833	0.844	1.000
Pungent	8	1.78	3	0.625	0.625	0.633	0.625	0.619	0.938

Thresholds denote the final taste-specific adaptive Weak/Strong cutoffs used for model training and evaluation. Values were calculated independently within each taste category and rounded to two decimals.

#### External validation and interpretability

External validation was conducted on nine herbal samples, excluding the Sour and Pungent modalities due to their suboptimal training performance. A three-tier evaluation system was established: Correct, Boundary Correct (ground truth within ± 0.2 of the soft margin), and Incorrect. The model achieved a strict accuracy of 77.78% (7/9). Notably, the lenient accuracy was identical to the strict accuracy, as all sample intensity values fell outside the defined soft boundary regions.

**Fig. 13:**
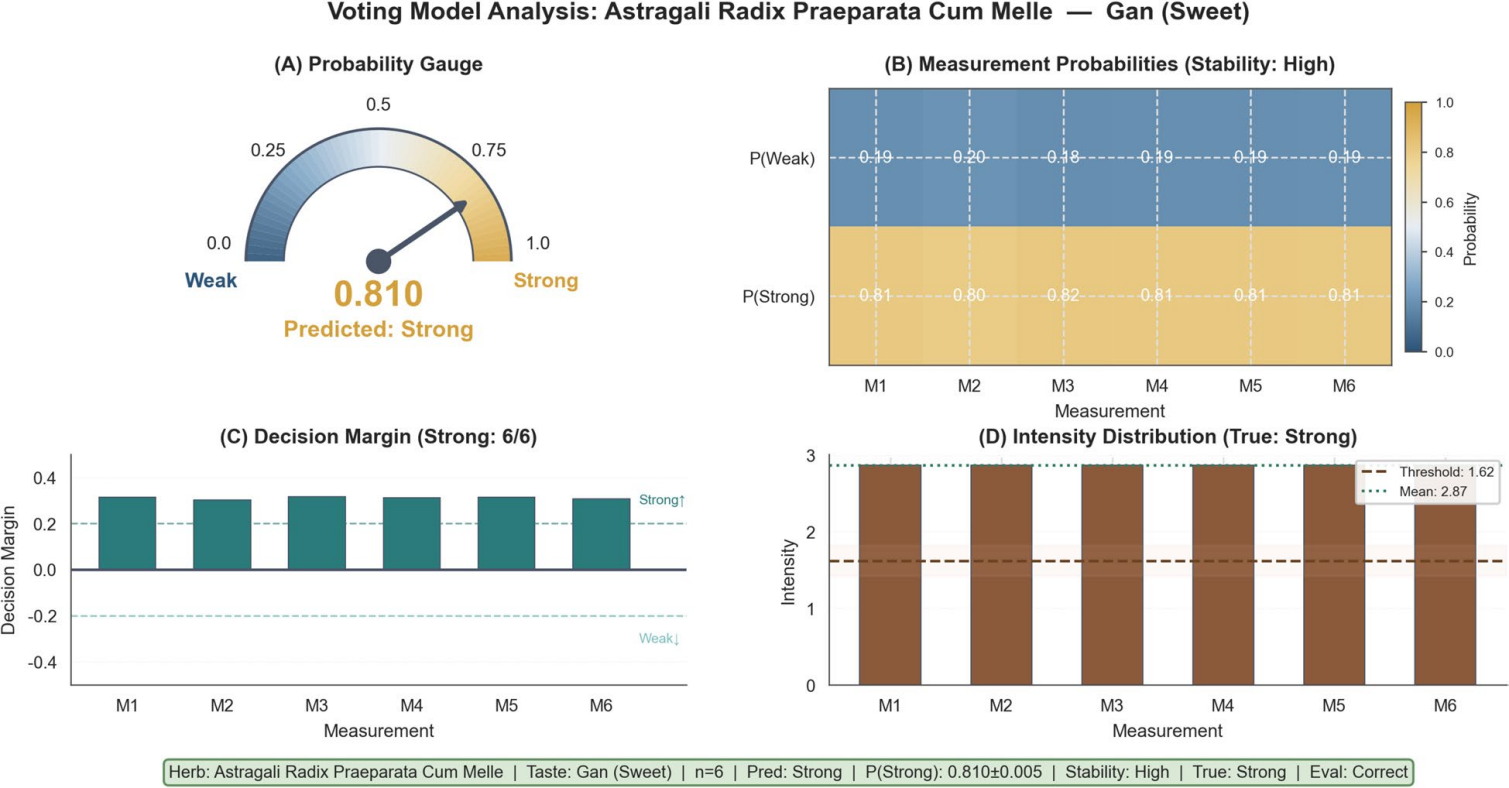
External validation of the Voting Ensemble model utilizing a representative case study of Astragali Radix Praeparata Cum Melle. Notes: The sample was accurately predicted as "Strong" with a high confidence probability (*P*_Strong_ = 0.810). The robustness of this classification is evidenced by the minimal standard deviation across six experimental replicates (σ = 0.005). The mean intensity (2.87) exceeded the decision threshold (1.62), indicating a clear discriminative margin and agreement with the ground-truth sensory label (True: Strong). **A** Overall probability gauge showing the final voting probability and predicted class. **B** Per-replicate measurement probabilities for Weak and Strong across six measurements (M1–M6). **C** Decision margins for each measurement (M1–M6), with all replicates voting Strong (6/6). **D** Intensity distribution across measurements with the decision threshold and mean indicated

Performance varied across taste modalities. The Bitter model demonstrated optimal performance (3/3, 100%) with a mean *P*(Strong) = 0.139, indicating high consistency in classifying "Weak" samples. The Salty model also achieved 100% accuracy (2/2) with maximal confidence (*P*(Strong) = 1.000). Conversely, the Sweet model achieved only 50% accuracy (2/4), misclassifying two "Weak" samples as "Strong". In a representative correct case, Astragali Radix Praeparata Cum Melle was predicted as "Strong" with *P*(Strong) = 0.810. The low standard deviation of probabilities across six replicates (σ = 0.005) and an intensity value (2.87) far exceeding the threshold (1.62) demonstrated strong alignment between the model prediction and sensory evaluation (Fig. 13).

**Fig. 14:**
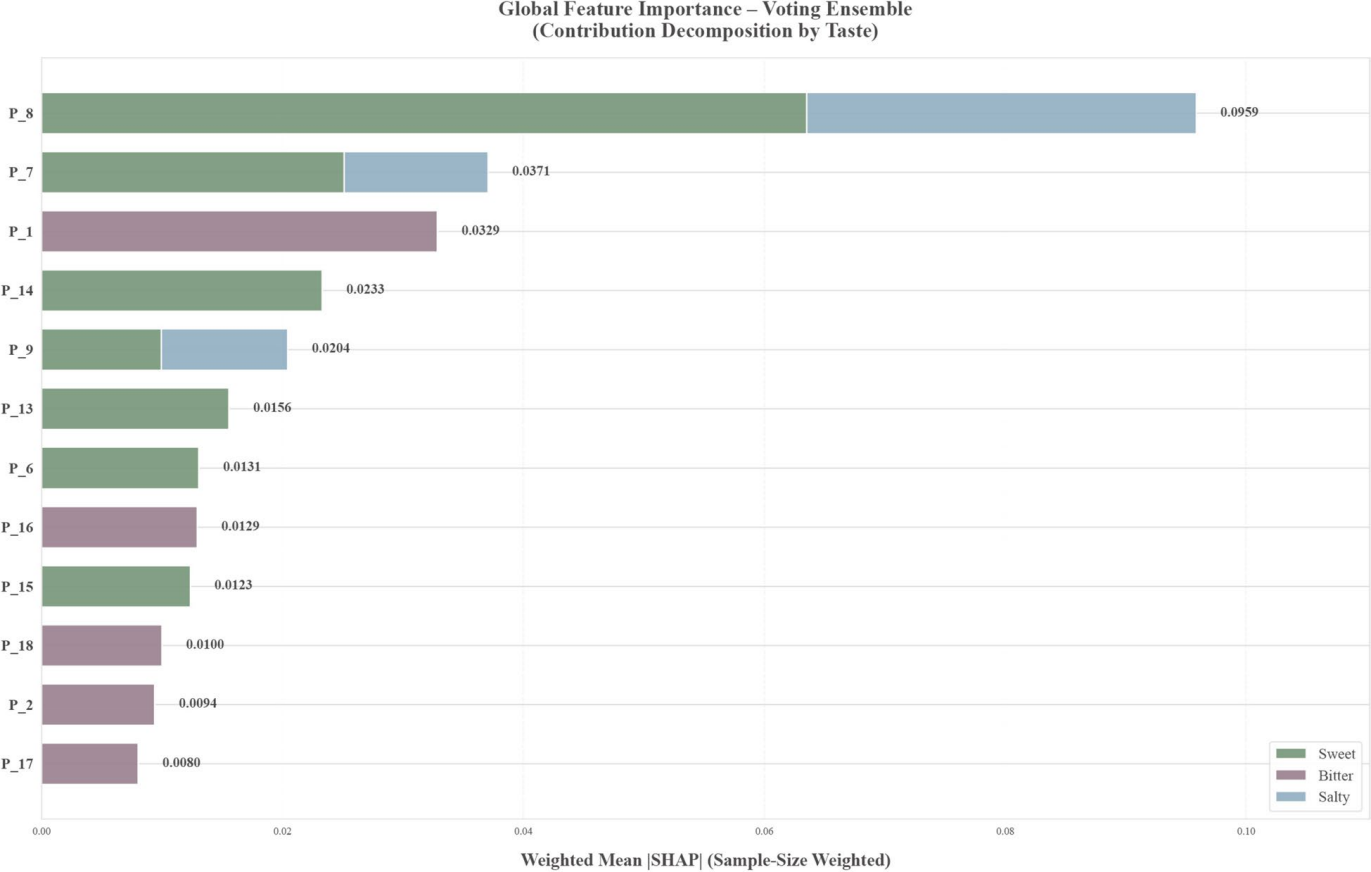
Global feature importance of the Voting ensemble model quantified by sample-size–weighted mean absolute SHAP values. Horizontal bars rank features (P_8–P_17) by their overall contribution magnitude (weighted mean |SHAP| on the evaluation set). Each bar is stacked by taste to show the decomposed contribution from Sweet (green), Bitter (purple), and Salty (blue); numbers at the bar ends denote the total weighted mean |SHAP| for each feature. Larger values indicate greater global influence on model predictions

SHAP analysis of the intensity models elucidated modality-specific feature importance patterns (Fig. 14). P_8 dominated predictions for Sweet (mean |SHAP|= 0.108) and Salty (0.259) tastes, indicating that this channel’s cross-sensitive response pattern carries the strongest discriminative information for these modalities. P_1 emerged as the most important feature for Bitter taste intensity prediction (0.115), a statistical pattern that may relate to the alkaloid-rich profiles of bitter herbs but requires electrode-level validation to establish a chemical basis.

**Fig. 15:**
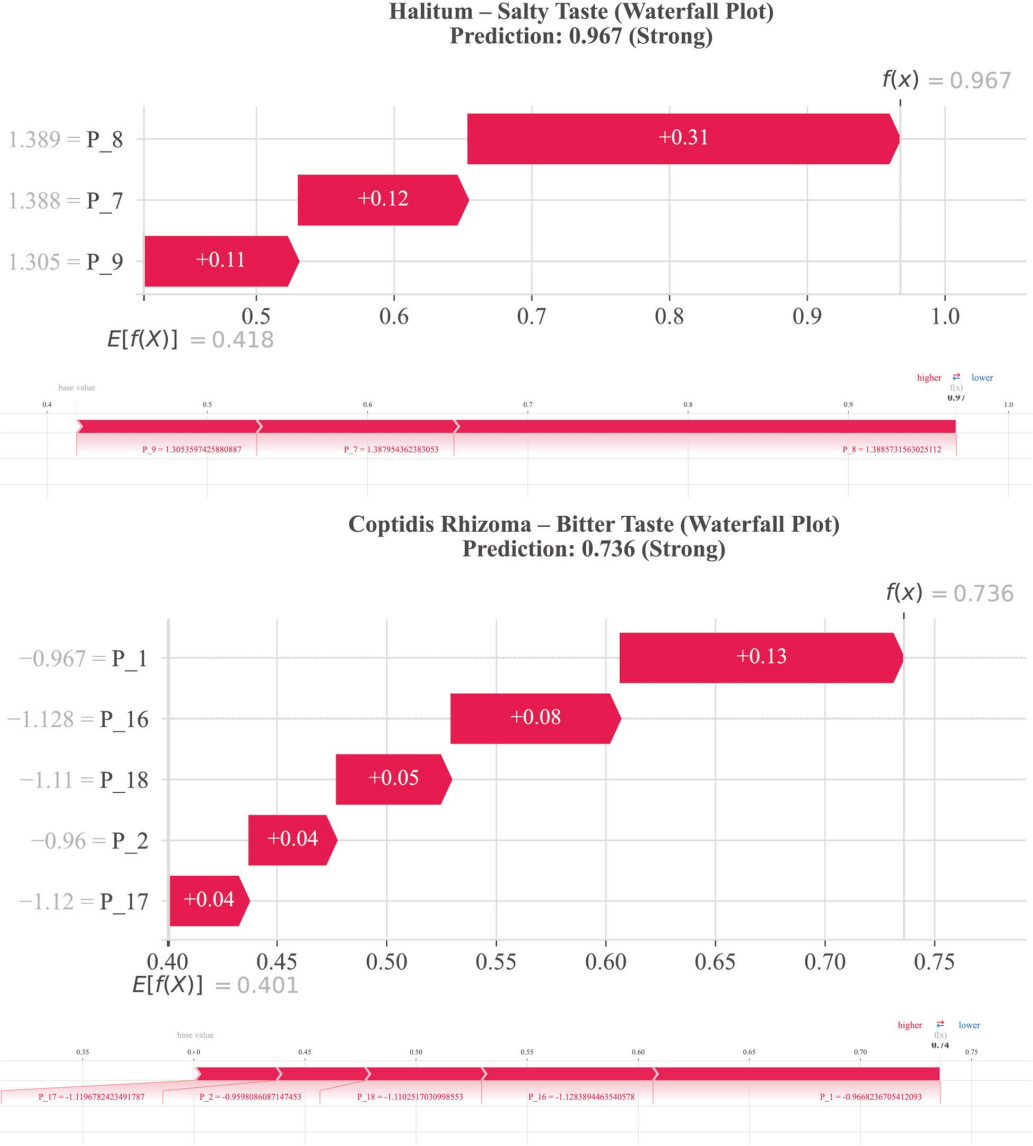
Individual-level feature attribution patterns revealed by SHAP waterfall plots using Halitum and Coptidis Rhizoma as representative herbs.

Sample-level waterfall plots for representative herbs corroborated these interpretable predictions (Fig. 15). Halitum (Salty, Intensity = 2.84) achieved the highest prediction probability (0.967); strong positive contributions from P_8 (+ 0.31), P_7 (+ 0.12), and P_9 (+ 0.11) were consistent with the high ionic conductivity expected from mineral salts acting on electrodes. Similarly, the bitter intensity prediction for Coptidis Rhizoma was primarily driven by P_1 (+ 0.13); while this statistical association is congruent with the alkaloid-rich chemical profile of this herb, the electrode-level mechanism underlying this response remains to be elucidated.

## Discussion

### Contemporary context of TCM five-flavor: from empirical categories to reproducible “instrumental sensory representations”

The “Five Flavors” of TCM—Pungent, Sour, Sweet, Bitter, and Salty—originate from centuries of empirical clinical practice and form a foundational component of TCM property theory. Modern sensory and analytical science aims to operationalize these descriptors into reproducible, quantitative representations to support quality control and cross-laboratory comparability. Recent studies have applied pattern recognition, chemometrics, and instrument-assisted sensory technologies, including E-tongues, to generate stable “taste fingerprints” mapped to sensory attributes or quality control (QC) endpoints via multivariate modeling [57, 58]. In this study, we frame Five Flavor identification as an instrumental sensory representation problem: E-tongue signals are captured and analyzed using heterogeneous ensemble models, with SHAP-based feature attribution providing data-driven hypotheses for further chemical and electrode-level validation, rather than mechanistic explanation.

### Key challenges: sensory consistency, complex matrix effects, and concept alignment

Human sensory evaluation remains the closest approximation to real-world taste, but its reproducibility is limited by inter-rater variability, physiological differences, and methodological biases. In our study, coefficients of variation ranged from 25.1% (Pungent) to 64.1% (Salty), indicating substantial inter-rater variability. Concept alignment is further complicated by chemically heterogeneous sensations, such as pungency, which cannot be fully captured by classical gustatory proxies. Instrumental measurements, including E-tongues, face additional challenges: multi-component matrices can induce competitive adsorption, masking, and non-linear sensor responses, while sensor drift, temperature effects, carryover, and cross-contamination threaten long-term stability [21, 59, 60]. These factors underscore the need for protocol standardization, drift correction, and calibration-update strategies. Accordingly, sensory labels should be treated as noisy, instrument-correlated proxies rather than definitive mechanistic truth.

### Modeling trajectory: architectural evolution driven by error diagnosis

Our modeling architecture evolved to address practical challenges in sensor-array data, following a problem-redefinition approach recommended for E-tongue analytics. Early TCM taste studies often focused on single taste dimensions, using conventional chemometrics or single learners (PLS/DA, DA, SVM/LS-SVM) under protocol-optimized conditions [61, 62]. These studies highlight a common risk: replicate measurements can inflate performance if not properly handled.

To mitigate such issues, we applied group-aware data splitting (StratifiedGroupKFold) to prevent information leakage and used cross-validated threshold adjustment to correct for class imbalance [63]. A unified multi-class framework proved structurally mismatched to our data due to differing flavor separability; hence, we adopted One-vs-Rest decomposition with flavor-specific classifiers. In the fusion layer, augmented meta-features (threshold indicators, confidence gaps, entropy, and dispersion) captured higher-order patterns of consensus and divergence. SHAP analysis quantified statistical feature contributions to model predictions, providing testable, data-driven hypotheses without implying mechanistic or chemical causation [13].

### The shift in intensity modeling: from unstable regression to grading with soft boundaries

For flavor intensity modeling, we similarly followed a diagnose-then-redefine strategy. Initial attempts with heterogeneous stacking regression (XGBoost/LightGBM/CatBoost) showed clear overfitting and limited validation performance. Correlation analysis suggested that E-tongue features were only moderately associated with certain intensities (e.g., Sweet and Bitter) and weakly associated with others, while panel variability and outliers introduced additional noise. Collectively, this implied that under small-sample conditions, E-tongue signals may be more sensitive to ‘categorical discrimination’ (determining class membership) but appear insufficient to support the stable mapping required for a reliable ‘continuous intensity regression’.

This observation is consistent with prior bitterness-quantification attempts. Yaroshenko et al. compared E-tongue, HPLC–UV, and CE-UV approaches for instrumental bitterness assessment of TCM samples, illustrating both feasibility and the practical complexity of linking instrumental profiles to a sensory endpoint [18]. Lin et al. further demonstrated that regression models (including robust PLS) can evaluate bitterness intensity of TCM decoctions using E-tongue data, but also emphasized the sensitivity to outliers and data quality—an issue that becomes critical in small datasets [62].

Therefore, instead of forcing a continuous regression under high label noise and limited sample size, we redefined intensity as a binary grading task (Weak vs. Strong). We used six complementary methods to identify data-driven thresholds based on distribution structure. Recognizing perceptual continuity near thresholds, we introduced a soft-boundary mechanism (δ = 0.2): samples close to the threshold were assigned reduced confidence weights. This design respects the inherent fuzziness of intensity boundaries, reduces the impact of noisy labels near cutoffs, and improves calibration in practical deployment scenarios.

### Future perspectives: toward transferable standards and the interpretation-validation loop

This study provides a proof-of-concept framework for Five Flavor identification in an external validation set. Future work can extend single-label models to multi-label or primary/secondary flavor modeling, integrate drift monitoring with domain adaptation or transfer learning for cross-device stability, and combine E-tongue signals with LC–MS, NMR, or spectroscopic fingerprints to strengthen instrumental-sensory mapping. Liquid–gas complementary sensing, pairing E-tongue liquid-phase profiles with volatile-phase E-nose or headspace GC–MS data, offers scalable and operationally relevant enhancements. Finally, SHAP-based attributions should be treated as statistical hypotheses, guiding targeted chemical validation and gradually bridging the gap between instrumental data and mechanistic understanding.

### Limitations and advantages: an objective assessment

It is necessary to objectively acknowledge certain limitations of this study. First, the dataset is small and imbalanced, which constrains the statistical power of both flavor identification and intensity grading, and limits the granularity of stratified analyses. For population-bounded datasets of this nature, a priori sample size calculation is impractical because the number of pharmacopoeially recognized single-label herbs in each flavor category is itself limited; post-hoc assessment indicates that minority classes (Salty n = 7, Sour n = 7) fall below the sample sizes generally recommended for reliable cross-validated evaluation, and performance estimates for these categories should accordingly be interpreted as preliminary. The external validation set, though fully independent, contains few samples, and performance metrics—particularly for minority classes—should be interpreted with caution. Extension to a multi-label architecture and larger, balanced datasets is needed in future work. Second, sensor-level characterization is limited. While E-tongue responses provide reproducible instrumental fingerprints, the 18 sensor channels (P_1 to P_18) are not assigned specific chemical selectivity. Accordingly, SHAP attributions indicate statistical associations rather than mechanistic or causal relationships, and correlated channels may lead to substitutable feature importance. Third, model performance is affected by class imbalance and small-sample constraints. Minority classes, such as Pungent and Salty, exhibit lower recall, and certain intensity classifiers are effectively non-functional. These findings underscore the importance of cautious interpretation and the need for further validation before deployment. Finally, despite these limitations, the framework demonstrates a pathway for reproducible Five Flavor evaluation, integrating instrumental signals with ensemble modeling and post-hoc statistical attribution. The approach prioritizes data integrity (e.g., group-aware splitting) and interpretable hypotheses while avoiding overstatement of mechanistic conclusions, providing a basis for future methodological improvements and chemical validation studies.

## Conclusions

This study presents a proof-of-concept framework coupling E-tongue sensors with ensemble learning for TCM flavor classification, achieving moderate performance with flavor-dependent variation. SHAP-derived feature attributions generate testable hypotheses linking sensor response patterns to pharmacologically relevant chemical classes, though these reflect sensor-level correlations rather than chemical mechanisms. Independent electrochemical and chemical validation is required before mechanistic interpretation can be drawn. Broader application—including operational or pharmacopoeial use—awaits larger and more balanced datasets, cross-laboratory replication, and chemical corroboration.

## Supplementary Information

Additional file1 (TIF 3473 KB)

Additional file2 (TIF 3198 KB)

Additional file3 (TIF 3439 KB)

Additional file4 (TIF 3971 KB)

## Data Availability

The original contributions presented in this study are included in the article/Supplementary Materials. Further inquiries can be directed to the corresponding author.

## Abbreviations

CP: Pharmacopoeia of the People's Republic of China
TCM: Traditional Chinese medicine
TCMs: Traditional Chinese medicines
E-tongue: Electronic tongue
SHAP: SHapley Additive exPlanations
PCA: Principal component analysis
t-SNE: T-distributed stochastic neighbor embedding
UMAP: Uniform manifold approximation and projection
SMOTE: Synthetic minority over-sampling technique
LR: Logistic regression
NB: Naive Bayes
DT: Decision trees
GBDT: Gradient boosting decision tree
SVM: Support vector machine
RF: Random forest
XGBoost: EXtreme gradient boosting
LDA: Linear discriminant analysis
ROC: Receiver operating characteristic
AUC: Area under the curve
SC: Silhouette coefficient
TP: True positive
FP: False positive
FN: False negative
TN: True negative
QC: Quality control
TAS2Rs: Taste 2 receptors
ML: Machine learning

## Declarations

### Supplementary data

Supplementary material related to this article can be found in the online version, including Table S1 (Sample sources) and Table S2 (Intensity grading and qualitative descriptors for the five basic tastes). Figures S1–S4 show external validation of the stacking ensemble model using representative case series.
