# Supplementary figures and images for: An integrated machine learning framework for TCM five-flavor classification based on E-tongue profiling and SHAP analysis
Source: Chin Med. 2026 Jun 4;21:159. doi: 10.1186/s13020-026-01399-9 (PMC13235049; doi:10.1186/s13020-026-01399-9)

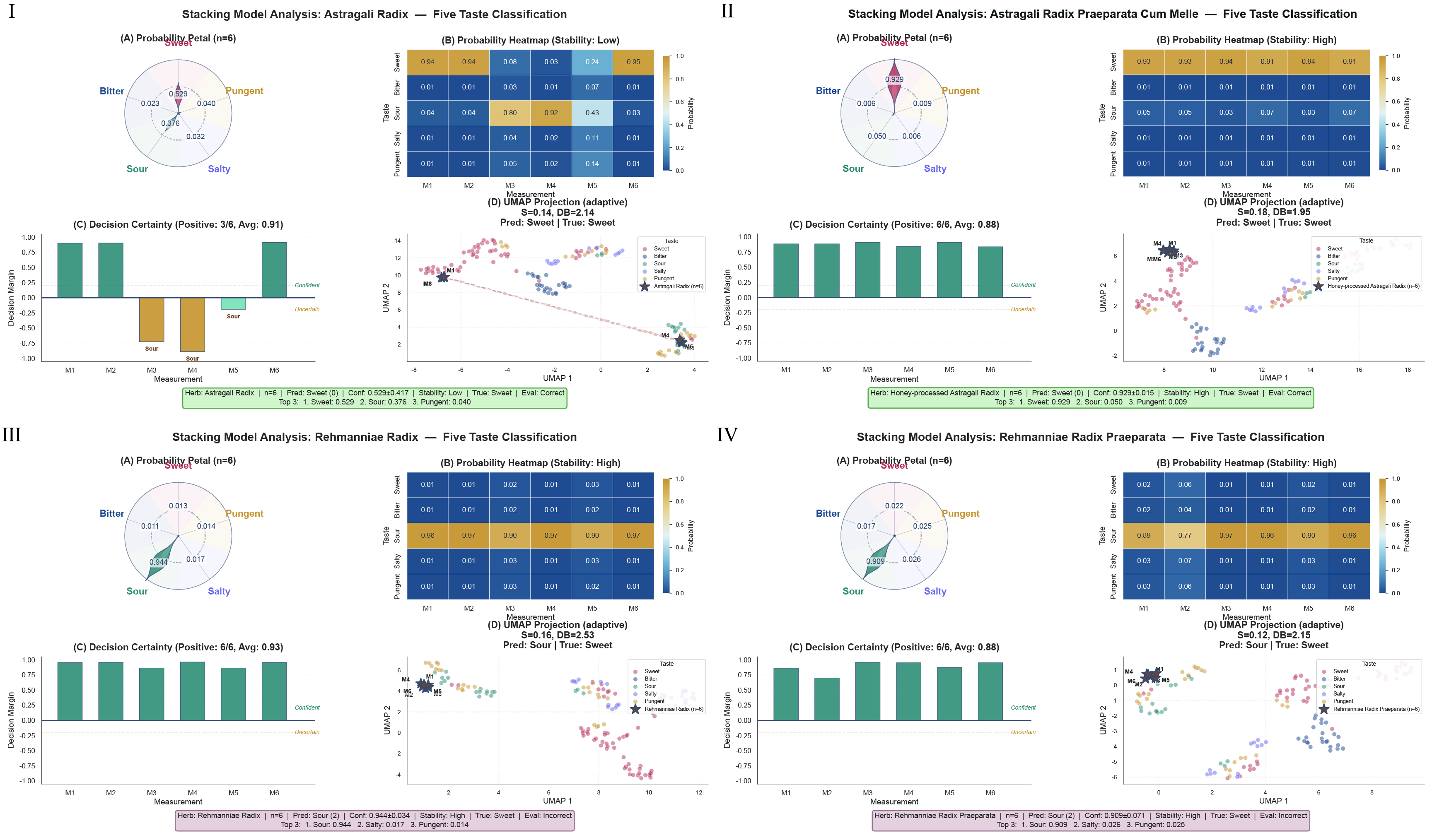

Supplement: Supplementary file 1 — Additional file1 (TIF 3473 KB) [file 13020_2026_1399_MOESM1_ESM.tif]

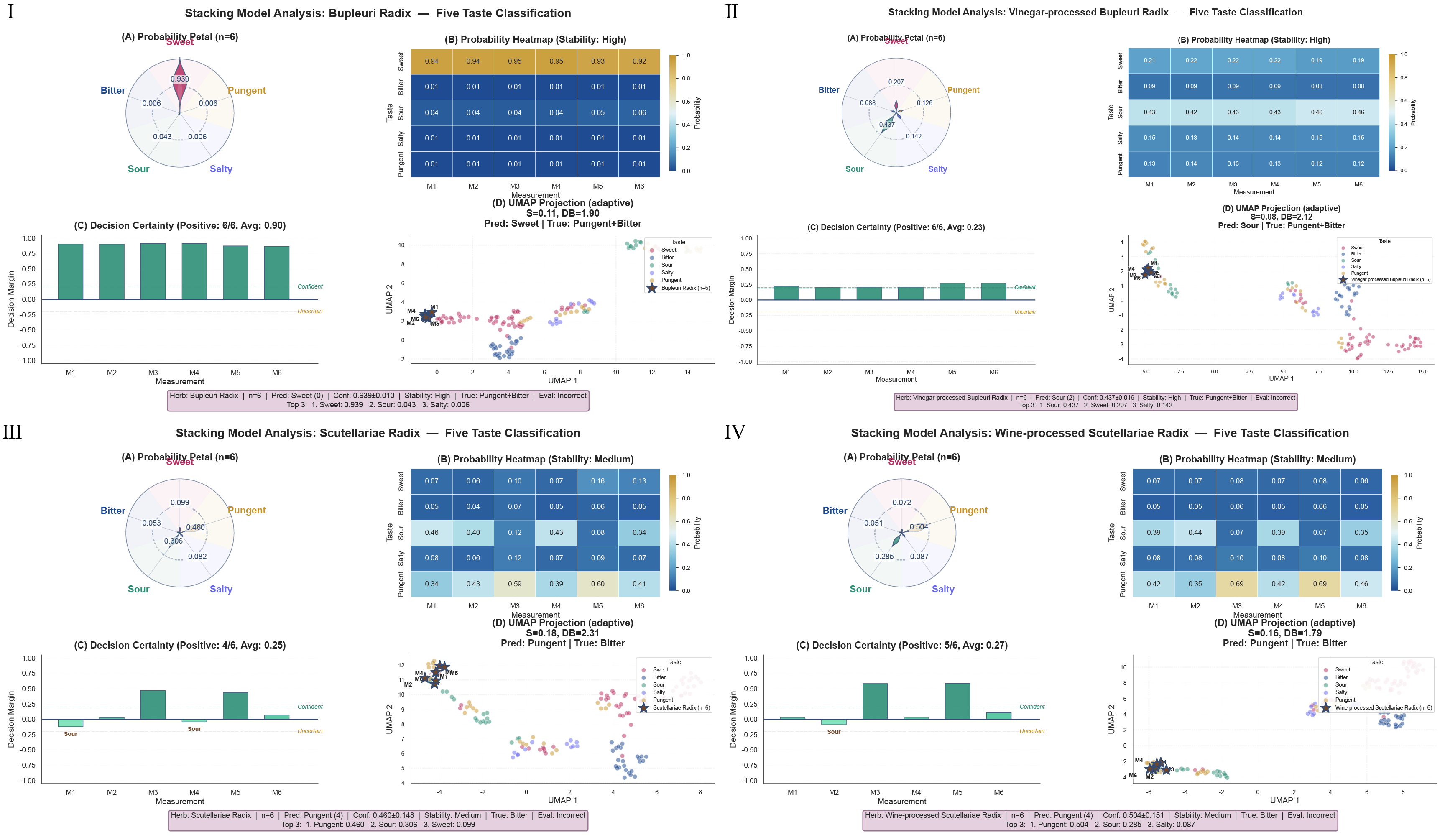

Supplement: Supplementary file 2 — Additional file2 (TIF 3198 KB) [file 13020_2026_1399_MOESM2_ESM.tif]

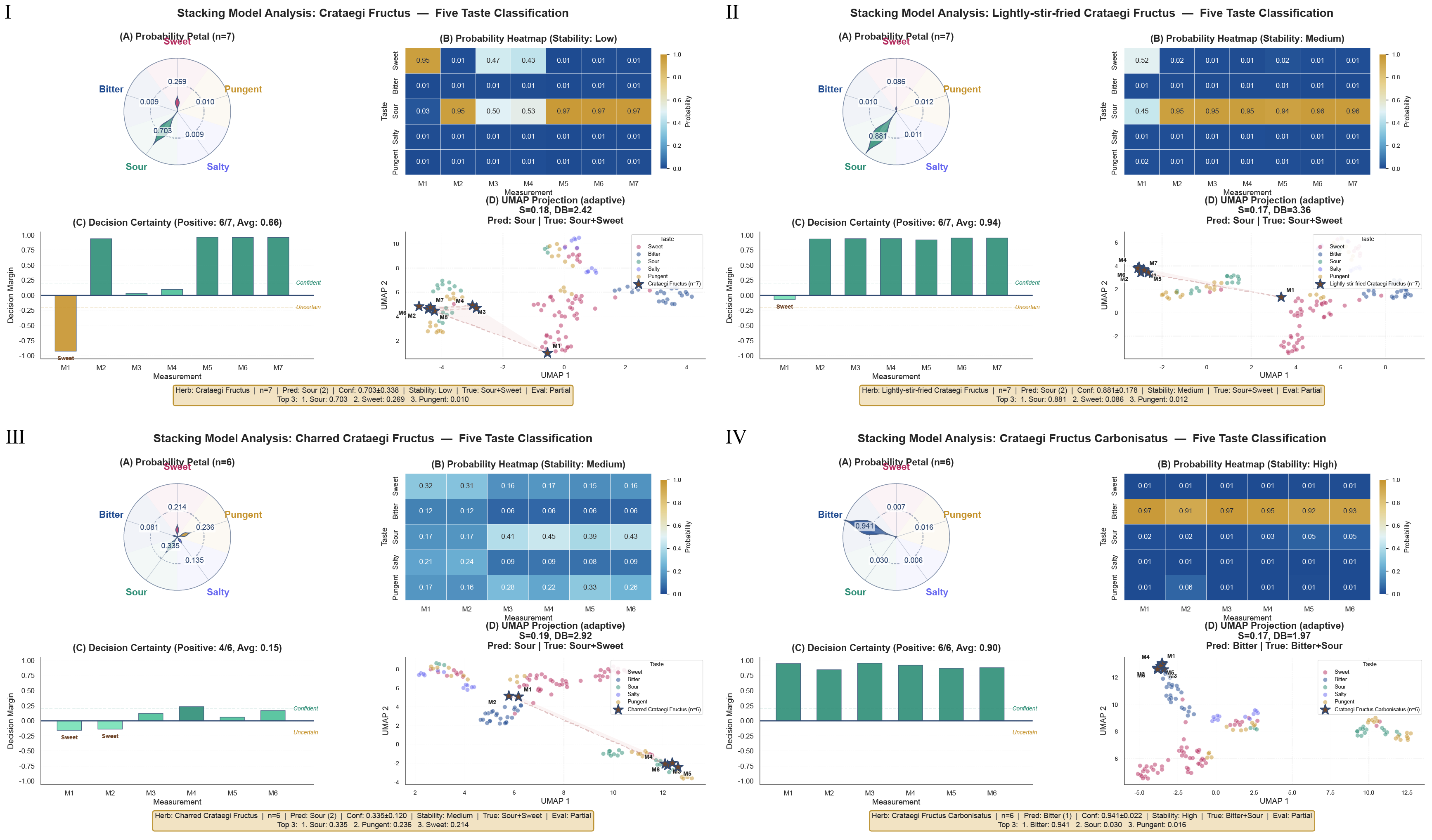

Supplement: Supplementary file 3 — Additional file3 (TIF 3439 KB) [file 13020_2026_1399_MOESM3_ESM.tif]

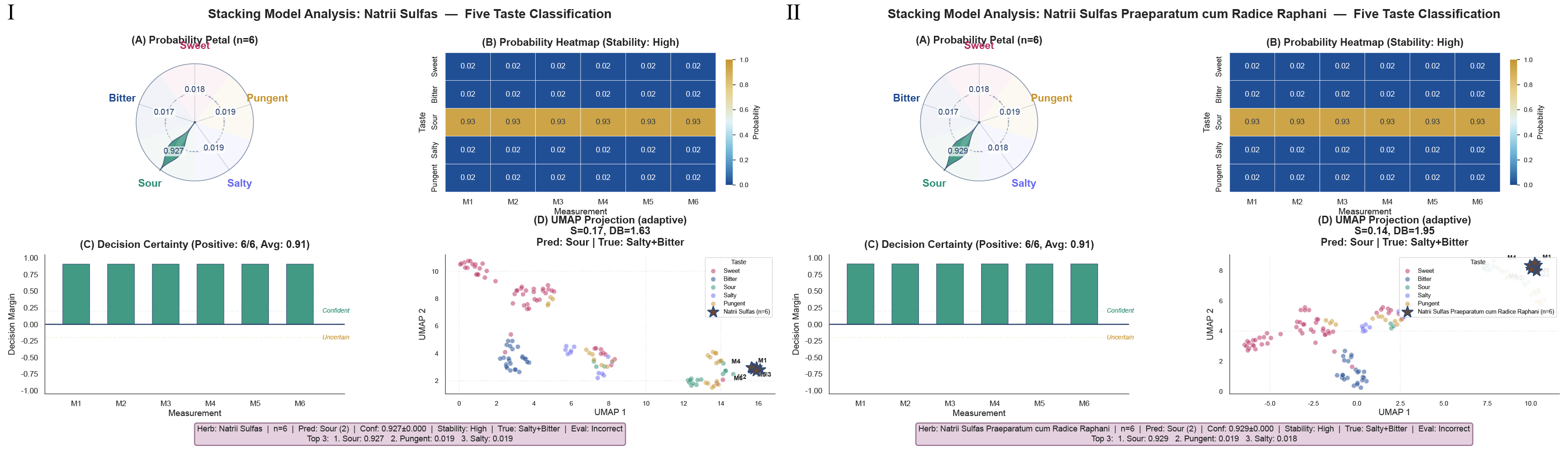

Supplement: Supplementary file 4 — Additional file4 (TIF 3971 KB) [file 13020_2026_1399_MOESM4_ESM.tif]
